# Powertrain configuration design for two mode power split hybrid electric vehicle

**DOI:** 10.1038/s41598-025-87378-w

**Published:** 2025-01-29

**Authors:** Tao Ke, Liangyi Nie, Andrés Kecskeméthy

**Affiliations:** 1 School of Mechanical and Electrical Engineering, Hubei Polytechnic University, Huangshi, 435003 China; 2 School of Mechanical Engineering and Electronic Information, China University of Geosciences (Wuhan), Wuhan, 430074 China; 3 Hubei Key Laboratory of Intelligent Convey Technology and Device, Huangshi, 435003 China; 4 Faculty of Engineering Sciences Institute of Mechatronics and System Dynamics, University of Duisburg-Essen, 47057 Duisburg, Germany

**Keywords:** Configuration design, Planetary gear train, Lever analogy, Design approach, Hybrid transmission, Mechanical engineering, Applied mathematics

## Abstract

Hybrid transmissions have attracted great attention in the automotive industry due to their energy-saving, low-emission properties, and have become a focus of research and development. This paper presents a new method to design the configuration of two mode power split hybrid transmission using the combination of the simple planetary gear trains (PGT). For this purpose, the hybrid transmission structure is divided into two substructures, which achieve different operation modes respectively. Then the output of one substructure is reconnected with the components of another substructure to obtain the complete hybrid transmission configuration. In addition, by adding additional shifting elements, the hybrid configurations can also realize the fixed gear mode as a traditional automatic transmission. As a result, 38 hybrid transmissions are obtained, and the kinematic analysis of a new configuration is performed for the subsequent optimization. Furthermore, using this decomposition and recombination method, the hybrid transmissions with multiple PGTs and different operation modes can be synthesized by changing the predefined modes.

## Introduction

Hybrid transmission is an effective approach to reduce fuel consumption and pollutant emissions, and it has become the focus of major automobile manufacturers’ research and development. The hybrid transmission with a power split configuration can achieve electrically variable transmission (EVT), which can significantly reduce fuel consumption and harmful gas emission of the engine. Consequently, such configurations have a significant advantage in hybrid transmissions. However, single-mode power split hybrid transmission cannot meet the requirements for a wide range of speed ratios. To solve these problems, the two mode power split hybrid power system was proposed, which combines two power split modes, and has better performance than the single-mode hybrid power system. The two mode power split configuration can possess the features of different power split modes and improve the transmission efficiency of the system and the performance of the vehicle.

**Fig. 1:**
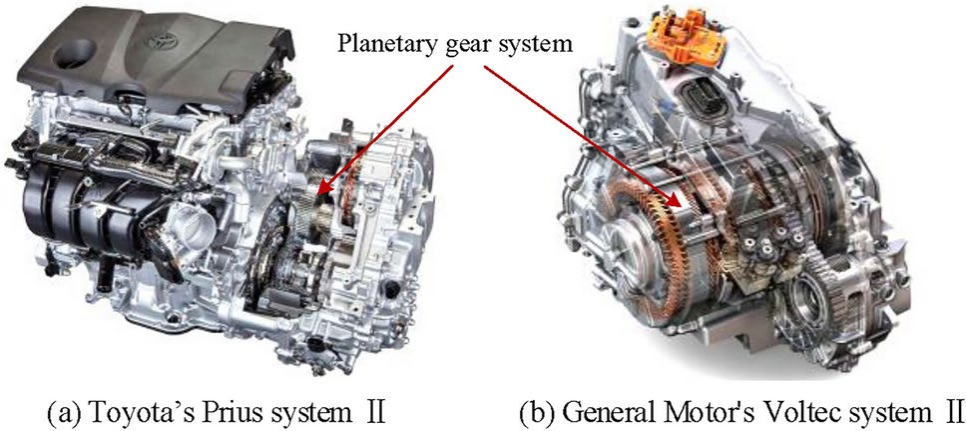
Toyota’s Prius system and GM’s Voltec system (Fig. 1 was taken by the corresponding author and granted to Springer Nature Limited for publication under a CC BY open access license).

**Fig. 2:**
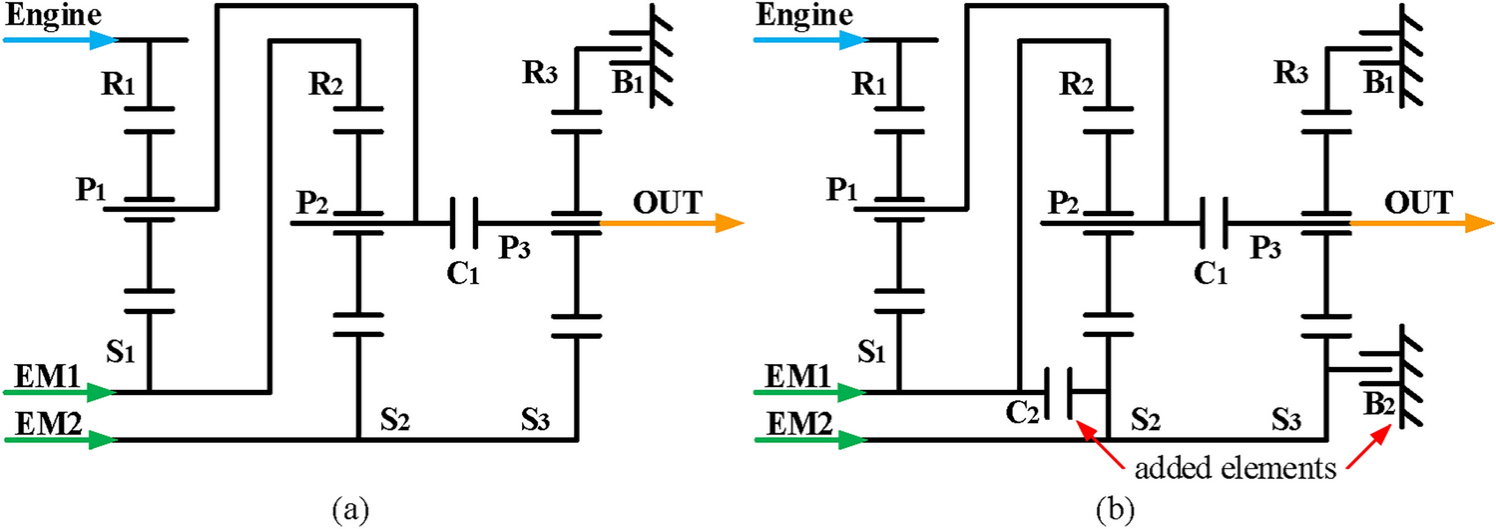
Two structure diagrams of GM two mode hybrid configurations.

A lot of work has been made on the configuration design of the hybrid transmission over the past few years. Two of the most classic hybrid transmission products are multi-generation Toyota’s Prius system^1,2^ and General Motors’ Voltec system^3,4^, as shown in Fig. 1 (Fig. 1 was taken by the corresponding author and granted to Springer Nature Limited for publication under a CC BY open access license). As for the two mode hybrid system, the two configurations of General Motors shown in Fig. 2 consisting of both a compound-split mode and an input-split mode are dominant. The configuration in Fig. 2b can realize four fixed speed ratios by adding two shifting elements based on the configuration in Fig. 2a. The four increased fixed-speed ratios are mainly used when the vehicle is driving under certain conditions, and the engine drives the whole vehicle independently with the fixed-speed ratio, which can greatly improve the transmission efficiency of the power system.

Many researchers have also proposed numerous suggestions and methods for the design of new hybrid transmission systems. For example, Tsai and his coworkers^5^ proposed a parallel hybrid transmission with five major modes, which is suitable for both rear-wheel and front-wheel drive vehicles. Behrooz^6^ introduced a novel hybrid powertrain system with two different power split modes, which can reduce transmission losses and improve efficiency. Chen^7^ proposed a novel EVT configuration with nine operation modes and described its mathematical modeling, analysis, and simulation. Esmail^8^ presented a parallel hybrid transmission system with only one electric machine and without any rotating clutches for mobile robots. Higuchi^9^ developed a two-motor plug-in hybrid system with three operation modes. Qin^10^ proposed a single-mode power split hybrid powertrain with three PGTs for tracked vehicles.

While creating new hybrid transmissions, some synthesis methods have also been proposed, the most widely applied of which is the lever analogy method. For example, Wang^11^ introduced a systematic analysis methodology to find out suitable schemes for power split transmissions from a variety of compound-split configurations based on the lever models. Zhuang^12^ proposed a searching and optimization methodology to explore the possible designs of multi-mode hybrid transmission with three PGTs. Liao^13^ presented an improved lever analogy method to simplify the analysis of automatic transmissions and hybrid powertrain transmissions. Ngo and Yan^14–16^ presented some design approaches to synthesize feasible configurations of hybrid transmissions based on the lever analogy method and obtained many feasible configurations. However, the lever method is not suitable for the automatic synthesis of complex configurations for its need to draw many lever diagrams in the synthesis process. In addition, there are some synthesis methods using other models. For example, Bayrak^17^ utilized a mathematical algorithm based on bond graphs to enumerate feasible hybrid powertrain architectures, and this method is generally to account for single and multi-mode hybrid transmission with different numbers of PGT. Xu^18^ presented a design approach based on matrix models to synthesize available configurations for dedicated hybrid transmission systems with multiple PGTs automatically. Cammalleri^19^ proposed a new matrix approach to identify the basic functional parameters from the transmission and upgraded a unified parametric model which is particularly promising to deploy in the hybrid electric powertrain. Deng^20^ proposed a systematic design method to synthesize the configuration of hybrid transmission based on the equivalent tree graph, which improves the design efficiency and reduces screen difficulty of the hybrid powertrain. Moreover, based on the principle of organic chemistry, they proposed a new design and screen method for a hybrid powertrain^21^. On the other hand, the performance analysis and evaluation of hybrid configuration is also the focus of research. Conlon^22^ developed a general analysis method to study the fuel economy and performance of the EVT mode. Zhang^23^ introduced a model for the operation simulation and optimization of multi-mode hybrid transmissions and proposed a novel hybrid transmission configuration with six operation modes. Zhu^24^ put forward a new multi-mode hybrid transmission with five power flow modes and studied its mathematical modeling and analysis. Cammalleri^25^ presented a novel model, which can allow engineers to prioritize the functionality and efficiency of the transmissions. Moreover, many patents also presented some new hybrid transmission configurations^26–31^.

Although the aforementioned works have contributed to the advancement of the analysis and design methods for hybrid transmissions, there are few literatures on the configuration synthesis problem of hybrid transmissions. Therefore, how to systematically synthesize the configurations of two mode power split hybrid transmissions as completely and efficiently as possible is still the main problem to be solved. Using the decomposition and recombination method, this paper provided a novel method for synthesizing the configuration of two mode power split hybrid transmission subject to design constraints, structural constraints, and required operation modes. Furthermore, this method can be used to synthesize hybrid transmissions with multiple PGTs and different operation modes.

The entire paper is arranged as follows. The design and structural constraints of a hybrid transmission are presented in Sect.  “Operation modes and structural constraints”. The required operation modes and their lever model are discussed in Sect. “Lever model of each operation mode”. The configuration design of two mode power split hybrid transmissions with three-PGT and two-PGT is conducted in Sect. “Configuration synthesis of two mode power split hybrid transmission”. The detailed kinematic analysis of a new two mode hybrid transmission is performed in Sect.  “Analysis of new two mode hybrid transmission configurations”. Finally, a summary and future research directions are presented in Sect. “Conclusion”.

## Operation modes and structural constraints

### Operation modes

The operation modes of hybrid transmission can greatly affect its performance. Based on the study of numerous existing hybrid transmission systems and research literature^11–19^, a two mode power split hybrid transmission should at least meet the following operation modes.

**Fig. 3:**
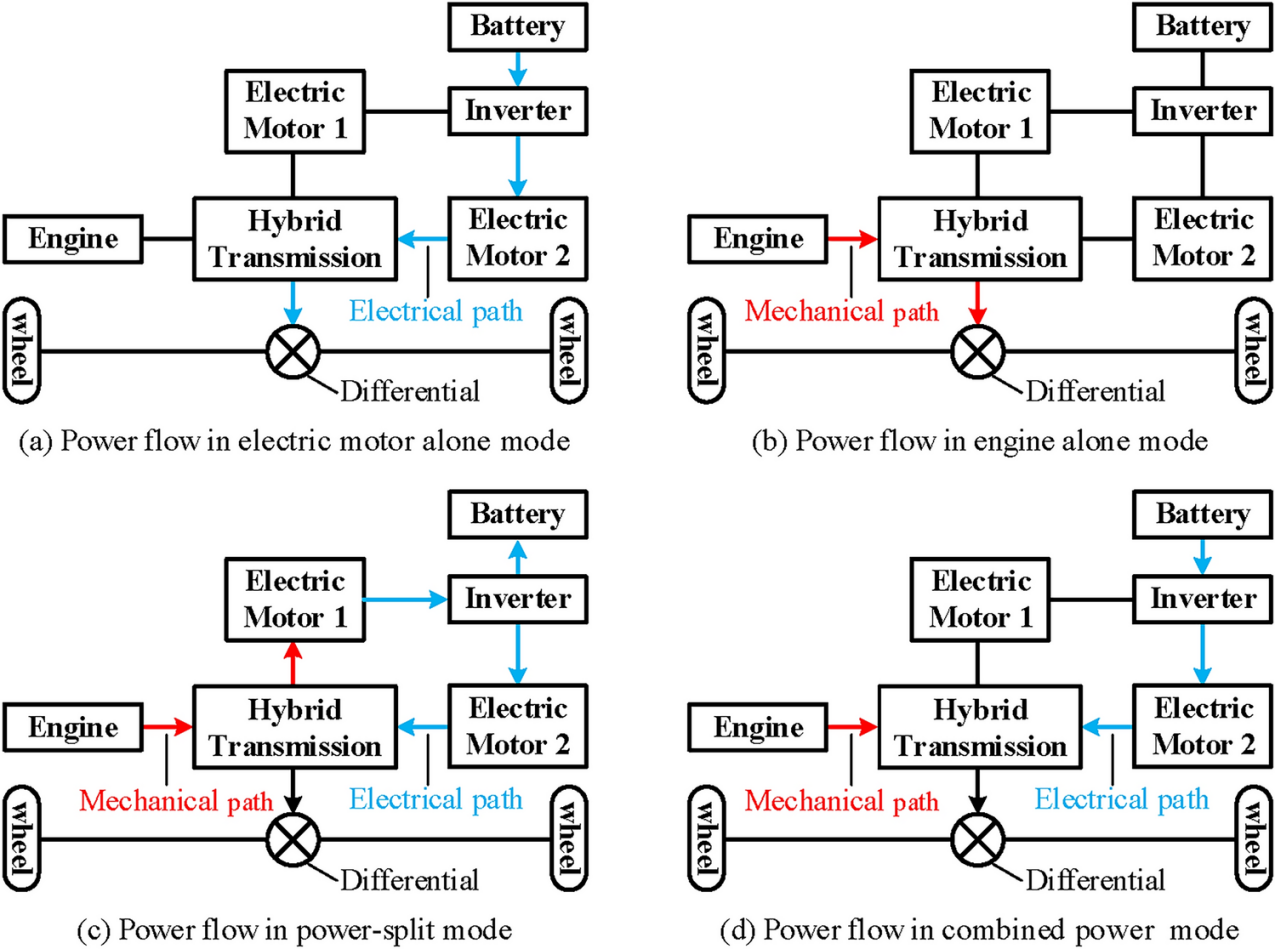
Power flow of each operation mode in hybrid transmission system.

1. Electric motor alone mode. Only one electric motor drives the vehicle, which usually occurs when driving at low speed or in situations with relatively low power demand. At this time, the battery is the only power source of the vehicle, and the energy flow of the vehicle is in the form of electrical power, as shown in Fig. 3a.
2. Engine alone mode. Only the engine drives the vehicle, which usually occurs when the power demand is light and can be met by the engine alone. At this time, the engine is the only power source of the vehicle, and the energy flow of the vehicle is in the form of mechanical power, as shown in Fig. 3b. In addition, for the engine alone mode, it is required to achieve at least three fixed speed ratios, including one under-drive, one direct-drive, and one over-drive. In particular, the hybrid system with three PGT is required to achieve two under-drive. Moreover, only one shifting element can be changed in the shifting process, just like a conventional automatic transmission.
3. Power split mode. In power split mode, the engine power is divided into two parts, one part is transferred to the output shaft by mechanical connection, the other part is converted to electric power by electric motor 1, and then to mechanical power by electric motor 2 to the output shaft, as shown in Fig. 3c. Specifically, there are three two mode power split configurations when input-split mode, output-split mode, and compound-split mode are coupled in pairs. But only the combination of input-split mode and compound-split mode has many applications. Therefore, it is required to have input-split mode and compound-split mode in this paper. In addition, switching between the two power split modes can only change one shifting element for ease of control.
4. Combined power mode. Both the engine and the electric motor drive the vehicle, which usually occurs when the power demand is relatively high and cannot be met by the engine or electric motor alone. At this time, the engine and battery are the power source of the vehicle, and the power flow of the hybrid transmission system is shown in Fig. 3d.
5. Regenerative braking mode. One or two electric motors can be turned into generators to regenerate electricity to charge the battery when the vehicle is braking. This mode can be switched from other modes during braking.

### Structural constraints

To distinguish the two electric machines, EM1 is used to denote the first electric machine mostly operated as a generator, and EM2 denotes the second electric machine mostly operated as a motor. To ensure the feasibility of the structure of hybrid transmission, a series of requirements are made for the arrangement of the engine, output shaft, and two electric machines.

**Fig. 4:**
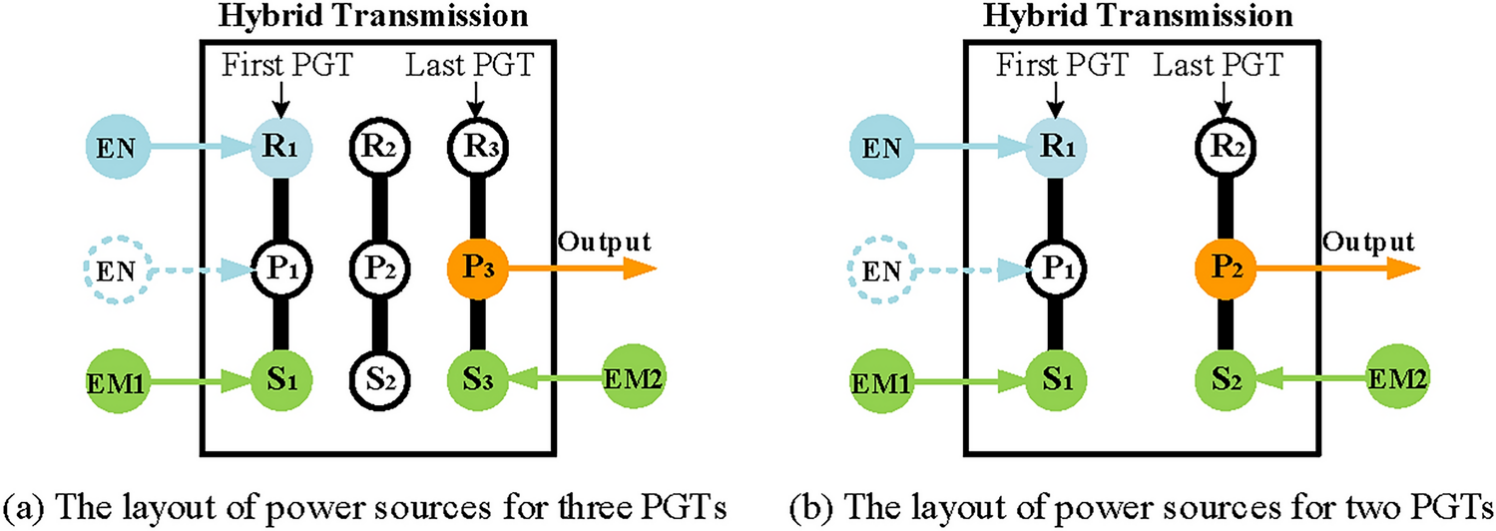
The layout of power sources for hybrid transmission.

1. As the fuel power input of the hybrid transmission, the engine is placed on the planetary carrier or ring gear of the first PGT to improve transmission efficiency and ensure output capacity^16^. For example, in multi-generation Toyota’s Prius system, their engines are placed on the planetary carrier of the first PGT, while in General Motors’ Voltec system, the engines are installed on the ring gear of the first PGT^18^. Therefore, the layout of the engine of the hybrid system with three PGTs discussed in this paper can be shown in Fig. 4. Here, the blue circle with EN denotes the engine, the yellow arrow with OUT denotes the output shaft of the hybrid transmission, and the green circle with EM1 and EM2 denote the two electric machines. Note that the specific structure of the PGTs is not given, because Fig. 4 only shows the layout of power sources. These specific structures of PGT will be determined in the subsequent synthesis process.
2. The EM1 is often used as a generator to generate electric power, especially in the power split mode. Therefore, to ensure the power generation performance of the EM1, the speed of EM1 is higher than that of the engine^16^. So, the EM1 is placed on the sun gear of the first PGT as shown in Fig. 4.
3. To ensure the output torque of the hybrid transmission, the output shaft of the whole transmission is placed on the planetary carrier of the last PGT as shown in Fig. 4.
4. The EM2 is often used as a motor to drive a vehicle, especially in the power split mode and electric motor alone mode. Therefore, to maximize the output torque of EM2, the EM2 is placed on the sun gear of the last PGT as shown in Fig. 4.
5. According to the existing configuration of hybrid transmissions, for the consideration of transmissions volume and shifting control, the number of brakes is no more than three and the number of clutches is no more than two.

## Lever model of each operation mode

The two mode power split hybrid transmissions designed in this paper are required to achieve input-split mode, compound-split mode, electric motor alone mode, and engine alone mode. The feasible configurations of each operation mode are separately analyzed below.

### Power split mode

In power split mode, the engine power is divided into two parts, one part is transferred to the output shaft by mechanical connection, the other part is converted to electric power by EM1, and then to mechanical power by EM2 to the output shaft. The addition of the electrical path makes the realization of EVT possible. Although there is energy loss in the energy conversion (mechanical energy to electrical energy to mechanical energy), the engine can be adjusted to work in a very efficient area by adjusting the ratio of two different power paths. Therefore, a very important point for a power split system is that the efficiency of some power transmission becomes lower locally, but the overall efficiency of the hybrid system is improved, namely, sacrificing local efficiency and improving global efficiency.

**Fig. 5:**
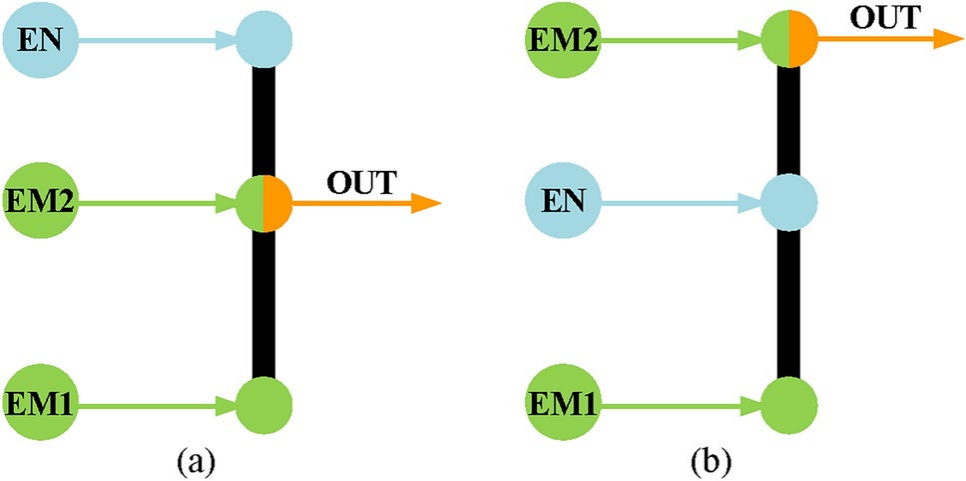
Two lever models of input-split mode.

**Fig. 6:**
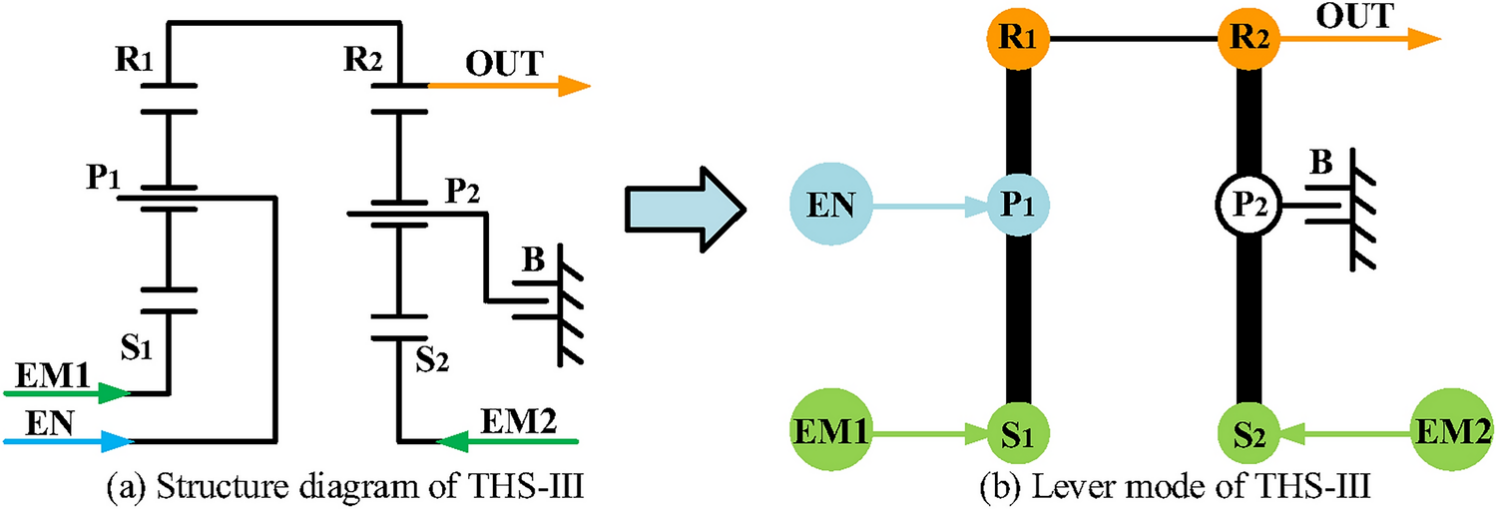
The structure diagrams and lever model of Toyota Prius THS-III.

For the input-split mode, two types of configuration design can provide it, and the lever models^22^ are shown in Fig. 5, in which the EM2 and output shaft (OUT) are connected to the same component of a simple PGT. However, for the hybrid transmission with multi-PGT, the EM2 can also be connected to the output shaft through a fixed transmission ratio. For example, Toyota installed a second PGT in the Prius THS-III^18^ through which the motor is connected to the output shaft and its structure diagram and lever model are shown in Fig. 6. Here, R_*i*_, P_*i*_, S_*i*_, (*i* = 1,2) and B represent the ring gear, planetary carrier, sun gear of *i* -PGT, and brake, respectively. It should be noted that the second planetary gear set has no power split function and only provides a fixed transmission ratio. Hence, because the hybrid transmission in this paper is composed of at least two PGTs, the EM2 and the output shaft are not connected to the same component, but through another PGT.

**Fig. 7:**
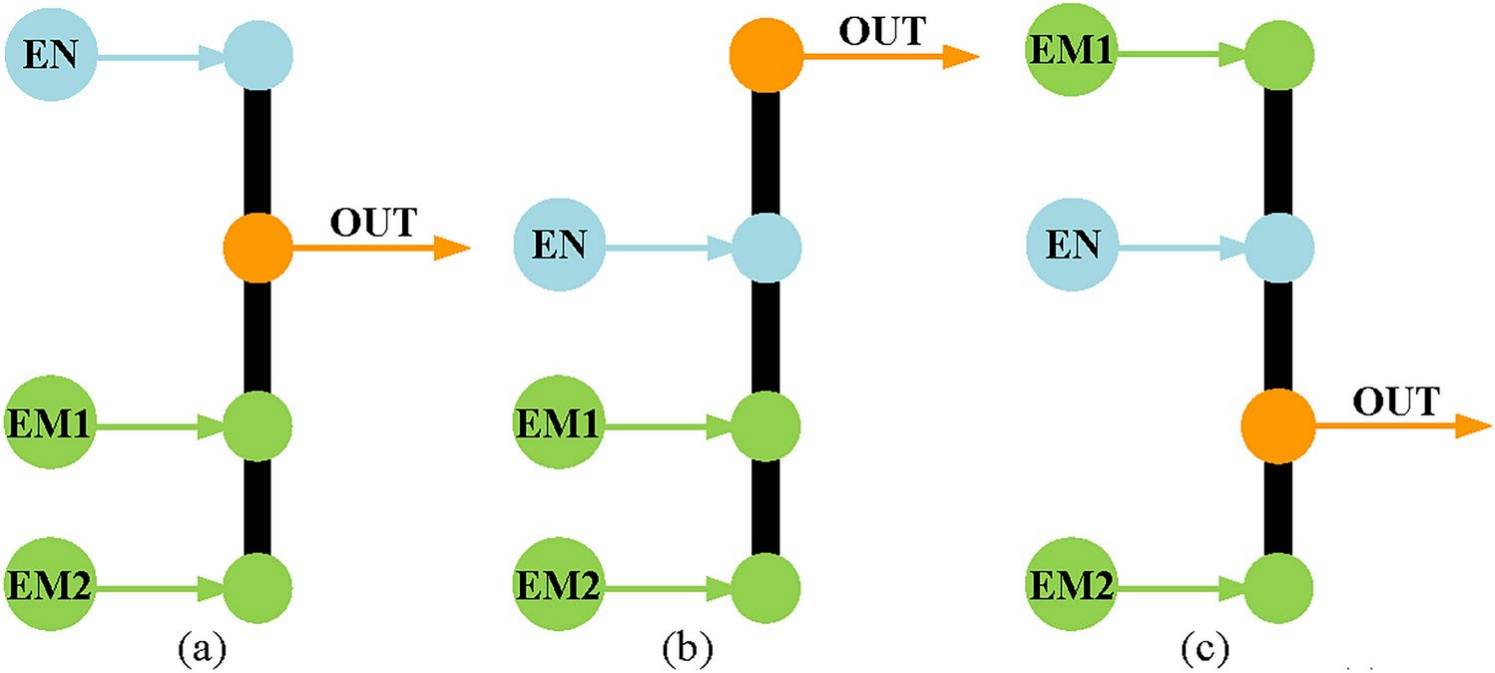
The lever models of compound-split mode.

As for compound-split mode, at least two PGTs are required, with the input-split mode in one PGT and the output-split mode in the other PGT. Three reasonable types of configuration design can provide compound-split mode, and the lever models^11^ are shown in Fig. 7. However, the configuration in Fig. 7a cannot take full advantage of the high-efficiency range between two mechanical points and it is not suitable for power split transmission. Moreover, the configuration in Fig. 7b divides more power into the electrical path, which will lead to relatively lower transmission efficiency at middle vehicle speed. Consequently, the relatively reasonable compound-split mode configuration in Fig. 7c is taken as the reference configuration in this paper.

### Electric motor alone mode

**Fig. 8:**
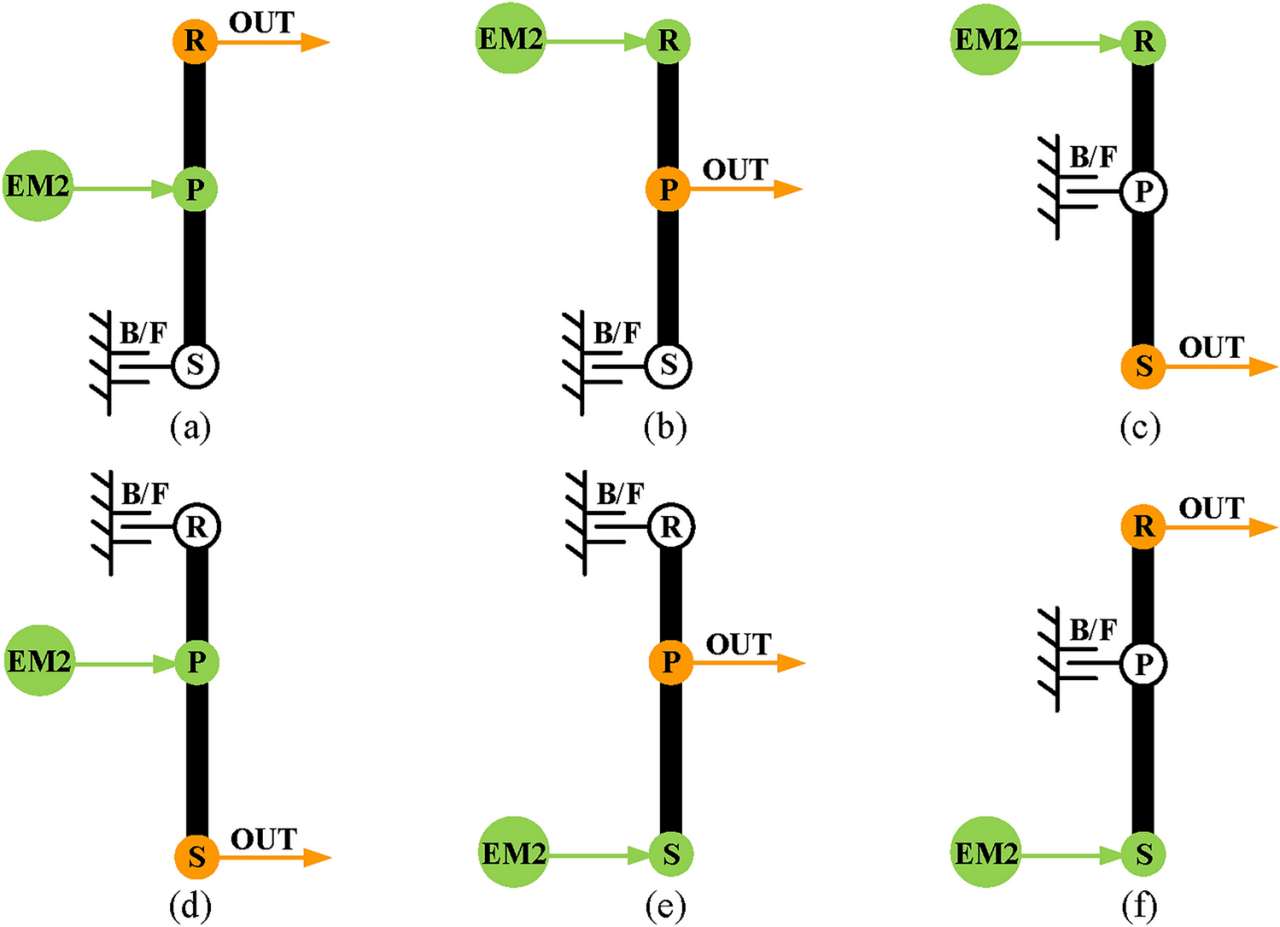
The lever models of electric motor alone mode.

In electric motor alone mode, EM2 is the only power input to the hybrid transmission. The EM2 and output shaft are placed on the same PGT (the last PGT) to ensure its output capacity. In the study based on existing designs of hybrid transmission, the electric motor alone mode has six types as shown in Fig. 8. Here the B/F denotes the component connected to the brake or fixed. However, according to the proposed structural constraints in Sect. 2.2 (Structural constraints). The EM2 can only be placed on the sun gear of the last PGT, and the output shaft is placed on the planetary carrier of the last PGT, so there is only one configuration for electric motor alone mode, as shown in Fig. 8e.

### Engine alone mode

In engine alone mode, only the engine drives the vehicle, and the hybrid transmission can work as a conventional automatic transmission by switching the operating mode of shifting elements. For the hybrid transmission with two-PGT, it is required to achieve at least one under-drive mode, one direct-drive mode, and one over-drive mode. But for the hybrid transmission with three-PGT, an additional under-drive mode is required because it adds one PGT. Since the engine alone mode does not require power from electric motors, it can be achieved by adding brakes and clutches to the configuration after the other operation modes have been implemented. However, it should be noted that only one shifting element changed during the shifting process of the different speed ratios of engine alone mode.

## Configuration synthesis of two mode power split hybrid transmission

**Fig. 9:**
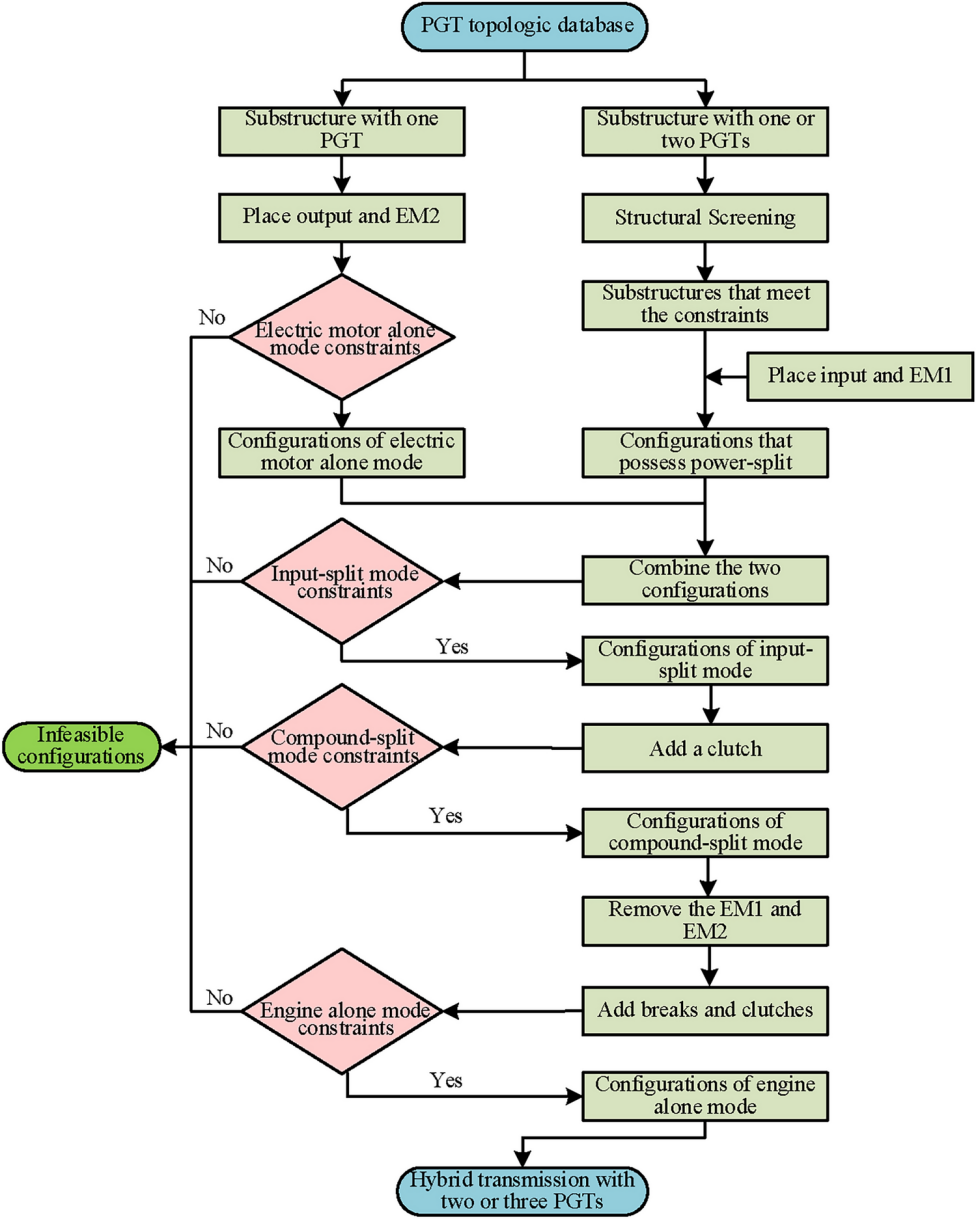
The main steps of the synthesis process.

Hybrid transmissions generally have two or three PGTs, so such hybrid transmissions are the object of this paper. After the previous analysis, the feasible configurations of each mode are obtained. The configuration of a two mode power split hybrid transmission is derived by combining the configurations of each operational mode. This forms the basis for the configuration synthesis of hybrid transmissions as presented in this paper. The synthesis process can be completed by the following steps, and the flow chart is shown in Fig. 9.

*Step 1*: Divide the structure of hybrid transmission into two simple substructures. Specifically, for a hybrid transmission with three-PGT, it can be regarded as the combination of a substructure with two-PGT and a substructure with one-PGT. And the hybrid transmission with two-PGT can be regarded as the combination of two substructures with one-PGT. These substructures are used to realize the electric motor alone mode, and the combination of these substructures can realize the input-split mode, compound-split mode, and engine alone mode. The specific process will be explained with an example in Section 4.1 (Configuration synthesis of hybrid transmission with three-PGT) and Section 4.2 (Configuration synthesis of hybrid transmission with two-PGT).

*Step 2*: Traverse all the substructures from our PGT atlas database^32–35^, and screen feasible substructures with two-PGT according to the structural constraints.

*Step 3*: Realize electric motor alone mode. This mode is implemented first because it can be achieved using a simple PGT. Therefore, select a substructure with one PGT and place the EM2 and output shaft on it according to the configuration obtained in Fig. 8e.

*Step 4*: Realize input-split modes. This mode can be achieved using two PGTs according to the analysis in Section 3.1 (Power split mode). Therefore, a PGT can be added to the configuration of electric motor alone mode to achieve input-split mode. Specifically, the substructure used to achieve electric motor alone mode can be used to provide a fixed transmission ratio and the power split function can be achieved by another substructure. Select the substructure with one or two PGTs and place the engine and EM1 according to the configuration obtained in Fig. 5 to achieve power split function. Then combine the two substructures by connecting one component (ring gear, planetary carrier, or sun gear) to complete input-split mode.

*Step 5*: Realize compound-split mode. This mode can be achieved by adding a clutch to the configuration of the input-split mode.

*Step 6*: Realize engine alone mode. The two electric machines are not work in this mode. Therefore, it can be achieved by removing electric machines from the configurations of compound-split mode. And then adding brakes and clutches according to the design requirements to realize fixed speed ratios.

## Configuration synthesis of hybrid transmission with three-PGT

**Fig. 10:**
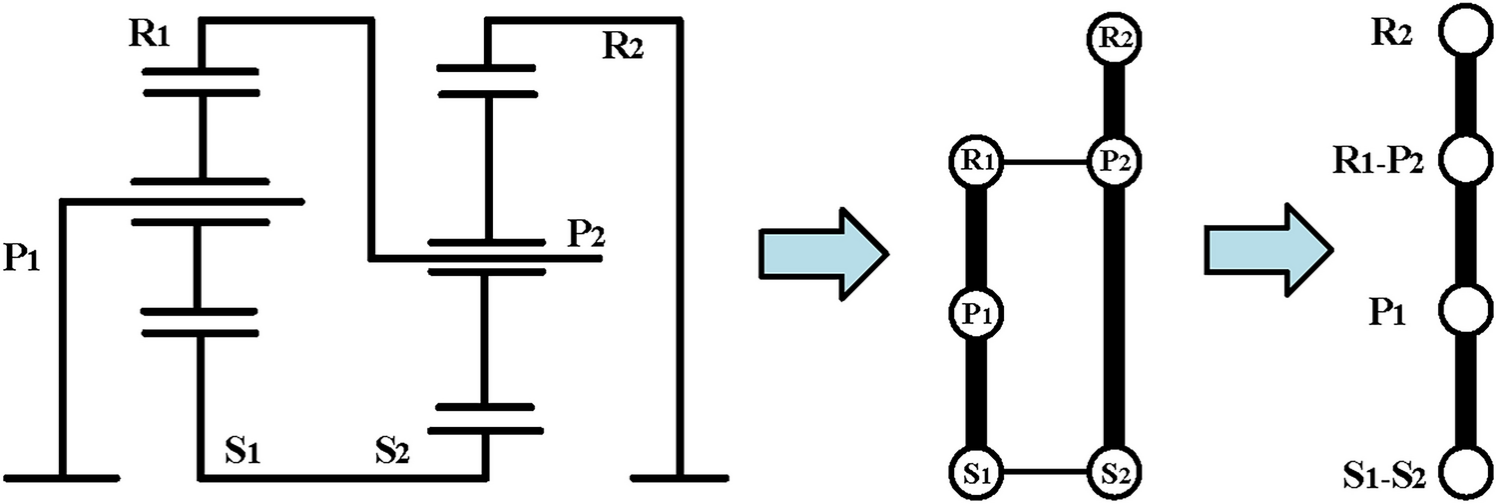
The function diagram and four-node lever model of the Simpson structure.

Firstly, for the configuration of hybrid transmission with three-PGT, it can be divided into two substructures at the “cut-link” according to the structural decomposition method^36^, one is the structure with two PGTs and the other is the structure with one PGT. For the substructure with one PGT which is called part I in this paper, a simple PGT is satisfied. The substructure with two PGTs is called part II in this paper. In our previous work, we screened the substructures that can be used for automatic transmission design from the topological graph library according to the structural constraints. The PGT used in both automatic transmissions and hybrid transmissions have the same structural constraints, so the constraints and screening results in Fig. 8 of reference^36^ can be used directly. For convenience, these substructures are labeled A to J in this paper. Given the increased complexity and lower transmission efficiency associated with the manufacturing process of the double-planet PGT, this paper only considers the single-planet PGT case. Taking the case of substructure A (Simpson structure) as part II as an example, the synthesis process will be introduced in detail. Figure 10 shows the function diagram and four-node lever model of the Simpson structure.

**Fig. 11:**
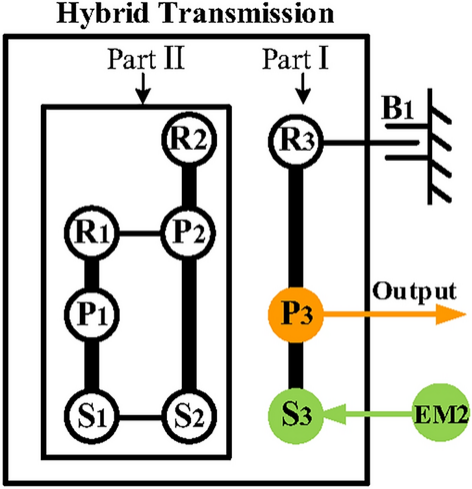
The electric motor alone configuration of a hybrid transmission system.

Secondly, realize electric motor alone mode. According to the previous analysis and the configuration in Fig. 8e, the output shaft is placed on the carrier of the last PGT, the EM2 is placed on the sun gear of the last PGT, and the B_1_ is placed on the ring gear the last PGT. Therefore, the electric motor alone mode configuration in a hybrid transmission system with three PGTs can be obtained as in Fig. 11.

**Fig. 12:**
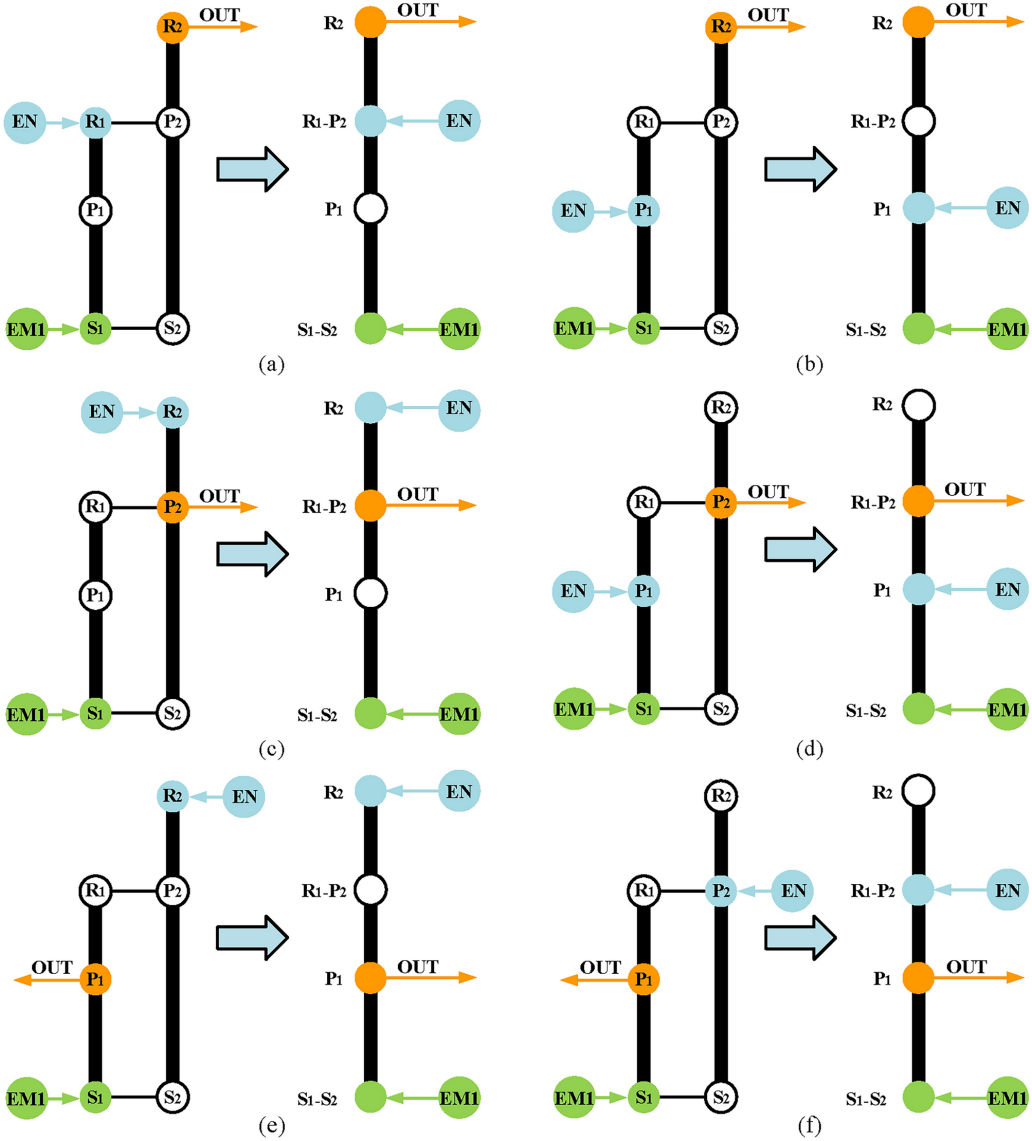
The six feasible input-split configurations for the Simpson structure.

**Fig. 13:**
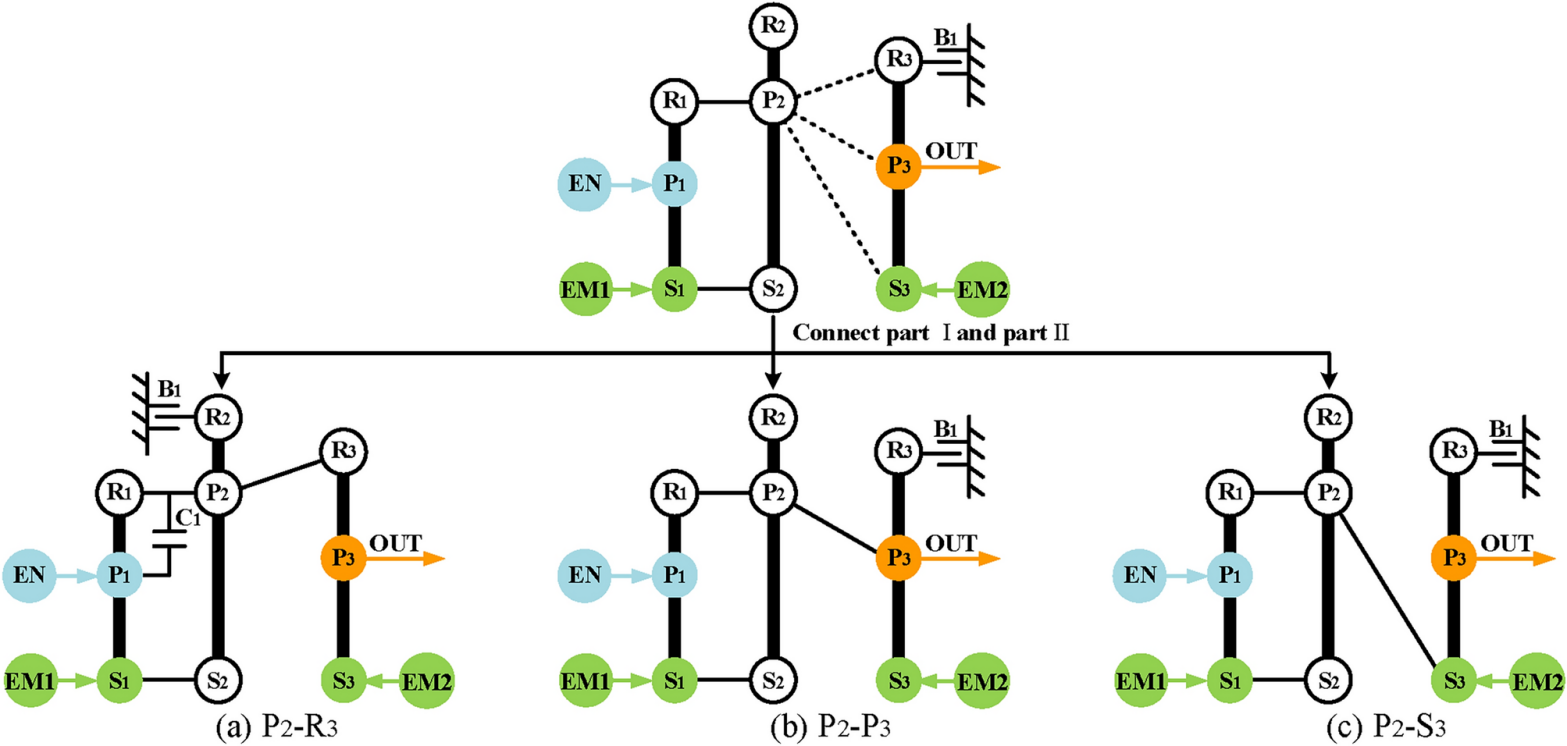
The three complete input-split modes for the configuration shown in Fig. 12d.

Thirdly, realize input-split mode. The power split function can be achieved by part II, and part I is used to provide a fixed speed ratio. According to the input-split mode shown in Fig. 5, there are six feasible configurations of input-split mode for Simpson structures as shown in Fig. 12. The next step is combining part I and part II to get complete configurations of input-split mode which has three PGTs. To be specific, it only needs to connect the output of part II shown in Fig. 12 and a component of part I shown in Fig. 11. Take the input-split configuration in Fig. 12d as an example, when P_2_ relates to the component of the part I, three feasible configurations with three-PGT are obtained and shown in Fig. 13. It should be noted that when the hybrid transmission is in the input-split mode, the brake B_1_ needs to work so that the part I can only transmit a fixed speed ratio and without the function of power split. However, when the output component (P_2_) is connected to the component with brake B_1_, the transmission of power from the engine and EM1 is prevented when B_1_ is working. Therefore, where brake B_1_ is placed needs to be transferred to part II. At this time, in order to meet the needs for R_3_ to be fixed in electric motor alone mode, namely R_3_ to be fixed when brake B_1_ is working, a clutch C_1_ is added to part II. When B_1_ and C_1_ are engaged at the same time, part II has zero degrees of freedom (DOF), as shown in Fig. 13a. Hence, when B_1_ and C_1_ are working, the R_3_ is fixed, and this state is the same as when B_1_ is placed directly on R_3_. As for the arrangement of C_1_, only the two disconnected components of part II can be placed, so that the DOF of part II is zero when C_1_ is working. There are six possible arrangements of C_1_, but the six arrangements serve the same purpose, only one is represented in this paper for simplicity.

**Fig. 14:**
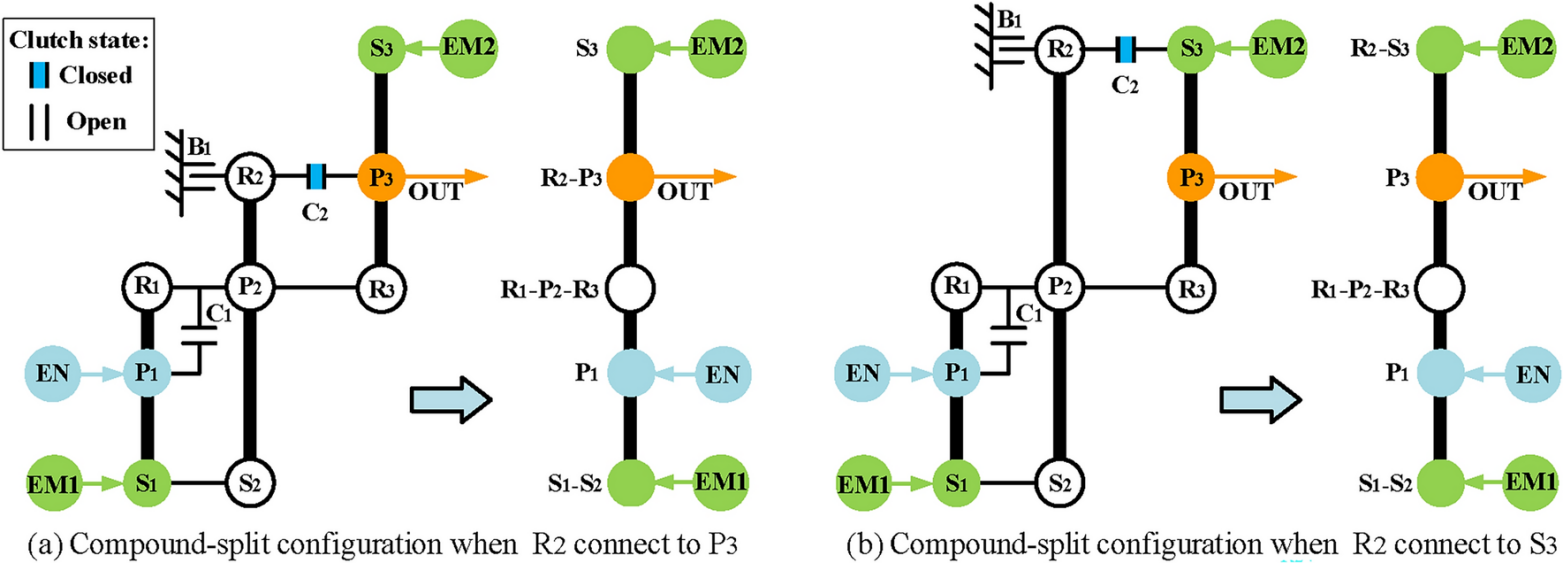
The two compound-split modes for the configuration shown in Fig. 13a.

Fourthly, realize compound-split mode. This mode can be achieved by adding a clutch C_2_ to the configurations of the input-split mode. As for the arrangement of C_2_, in order to reduce the structural complexity, C_2_ is specified to be added between the components of the second PGT and the third PGT. Take the configuration in Fig. 13a as an example, when the C_2_ is placed between the components in the second and third PGT, there are four possible ways (R_2_-P_3_, R_2_-S_3_, S_2_-P_3_, S_2_-S_3_), but only two configurations can realize compound-split mode shown in Figs. 14a and b.

**Fig. 15:**
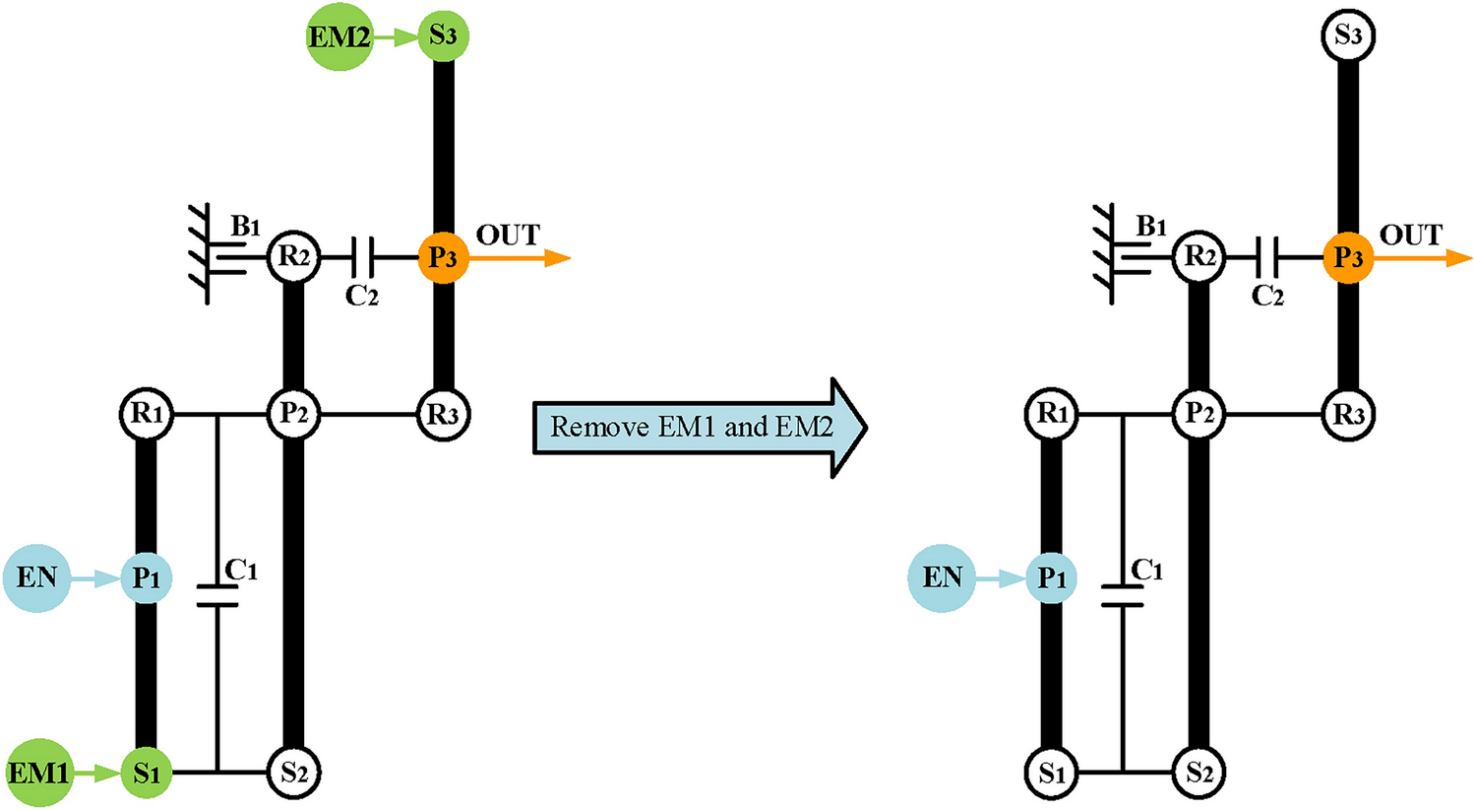
The configuration of Fig. 14a after removing EM1 and EM2.

Finally, realize engine alone mode. In this mode, only the engine drives the vehicle, and the hybrid transmission can work as a conventional automatic transmission, so the EM1 and EM2 can be removed when adding brakes and clutches. Take the configuration in Fig. 14a as an example, Fig. 15 shows its configuration after removing the EM1 and EM2. After removing the motors, the DOF of the PGT of the hybrid transmission is two, which requires two shifting elements to work to achieve the mode of fixed speed ratio. The traditional lever method is generally used in shift analysis of PGT with one-DOF. Hence, it is troublesome to use the traditional lever method to analyze the hybrid transmission with two-DOF. To solve this problem, the structural decomposition method proposed in our previous works^36^ is used to divide the structure of hybrid transmission into two substructures with one DOF.

**Fig. 16:**
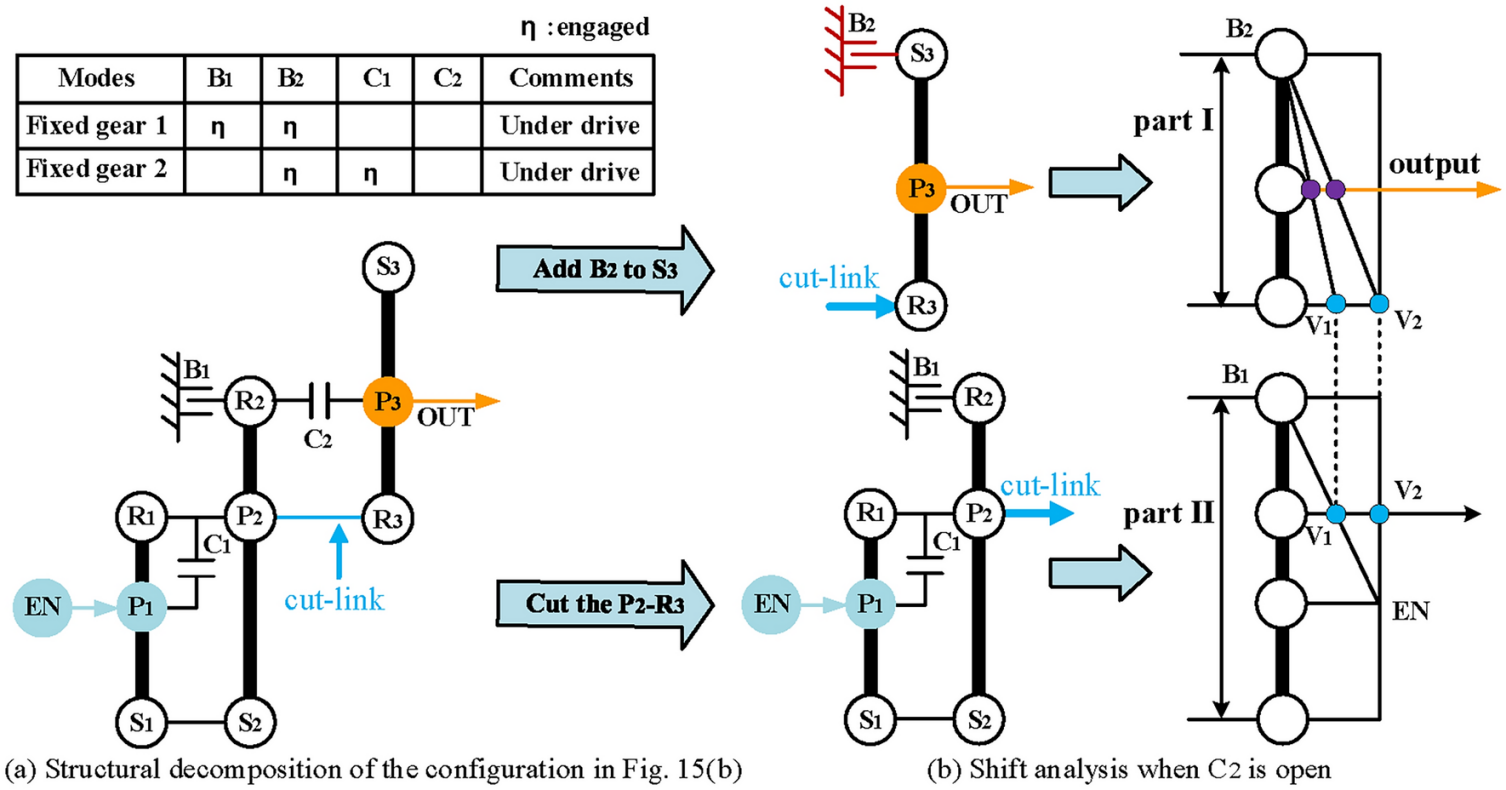
Structural decomposition and shift analysis of Fig. 15 when C_2_ is open.

When clutch C_2_ is open, the hybrid transmission has two DOFs, which requires structural decomposition to simplify its six-node lever model for shift analysis. Take the configuration in Fig. 15 as an example, the component P_2_-R_3_ can be chosen as the cut-link to divide the hybrid transmission into two parts with one DOF, as shown in Fig. 16a. The next step is to add shifting elements and determine the clutching sequence. The following constraints need to be noted when adding additional shifting elements:

1. Due to the constraints of the number of shifting elements, the clutches can no longer be added, and only two brakes can be added at most.
2. The additional brakes should not be placed on the component of the output shaft and engine to avoid affecting the power input and output.
3. Since the motor does not work in engine alone mode, the brake can be placed on the component of EM1 and EM2.
4. The placement of the brakes shall not interfere with other components.

In part II, two intermediate speeds (V_1_ and V_2_ in Fig. 16b) can be generated at the cut-link (P_2_-R_3_) by closing B_1_ and C_1_ respectively, which can be regarded as the input of part I. To generate the fixed output speed ratio at the output shaft (P_3_), a brake needs to be added to part I. The brakes B_2_ can be placed on the sun gear of the last PGT (S_3_) based on the above constraints. Consequently, two under-drive (the purple dots) are realized through the cooperation of C_1_, B_1,_ and B_2_, as shown in Fig. 16b.

**Fig. 17:**
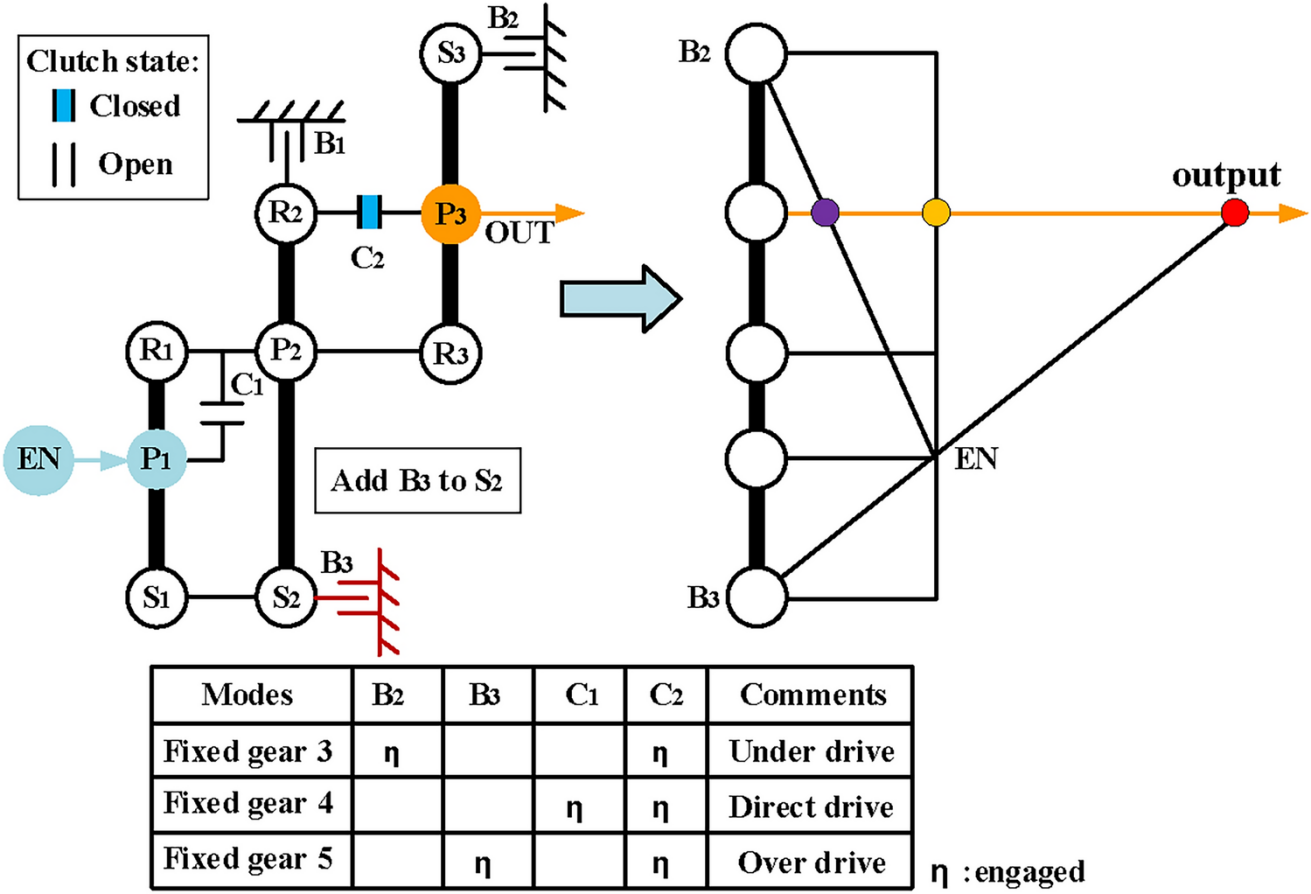
Shift analysis of Fig. 15 when C_2_ closed.

When C_2_ is closed, the hybrid transmission has one-DOF, the traditional lever method can be used in this case, and its five-node lever model is shown in Fig. 17. According to the shift analysis, when C_2_ is closed, an underdrive (the purple dot) and a direct drive (the orange dot) can be generated at the output shaft through the cooperation of B_2_ and C_1_, but there is no overdrive. However, according to the five-node lever model, the over-drive can be realized only by adding a brake at S_2_. Therefore, the brake B_3_ is placed on the S_2_, and three fixed-gears are generated through the cooperation of B_2_, B_3_, C_1,_ and C_2_, as shown in Fig. 17. The clutching sequence of the hybrid transmissions is shown in Table 1.

**Table 1:** The clutching sequence of the hybrid transmissions in Fig. 17.

Modes	B_1_	B_2_	B_3_	C_1_	C_2_	Comments
EV	●			●		EM2 alone work
Input-split	●					EVT
Compound-split					●	EVT
1st FG	●	●				Underdrive
2nd FG		●			●	Underdrive
3rt FG		●		●		Underdrive
4th FG				●	●	Direct drive
5th FG			●		●	Overdrive

**Fig. 18:**
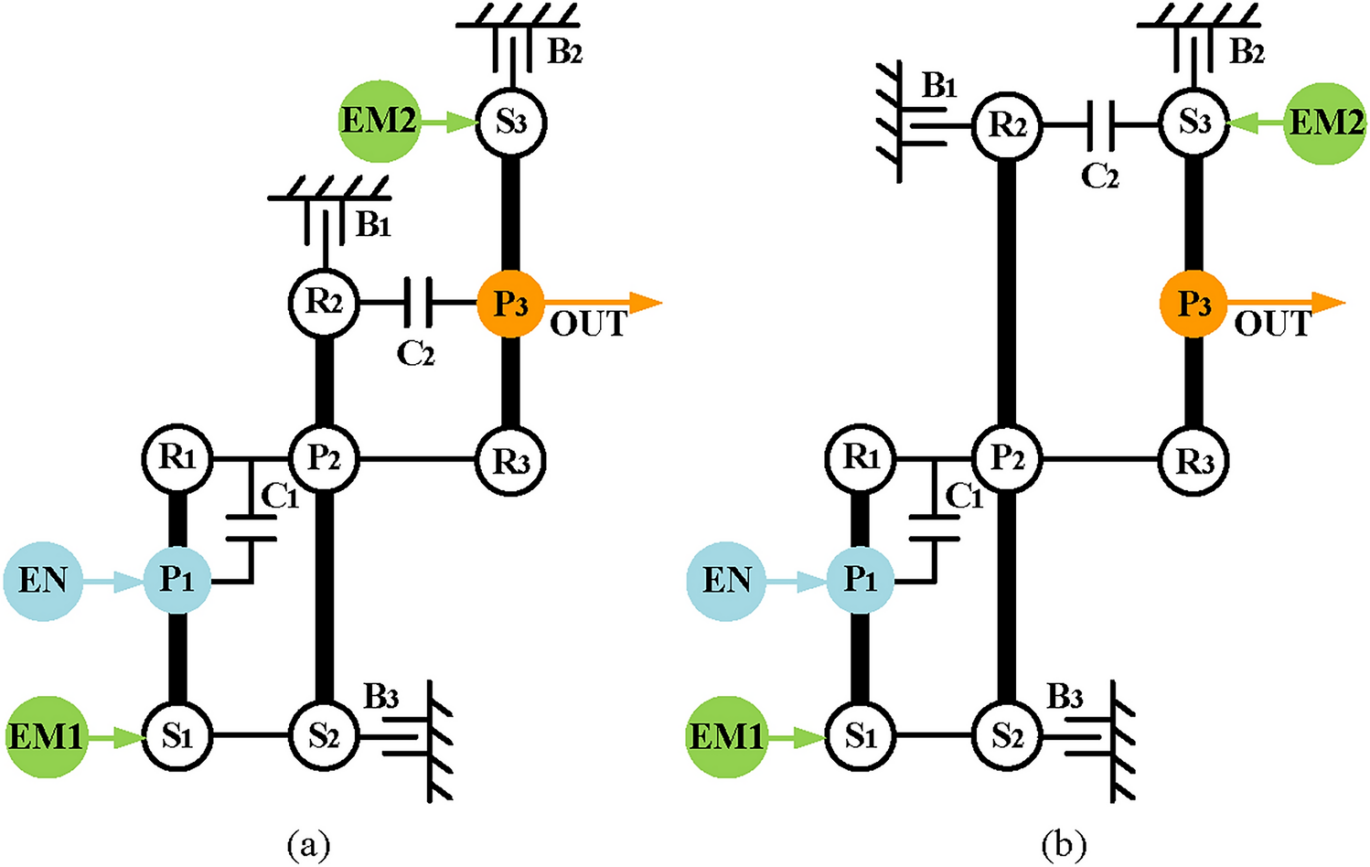
Two lever models of two mode configurations synthesized from the input-split configuration of Fig. 13a.

**Fig. 19:**
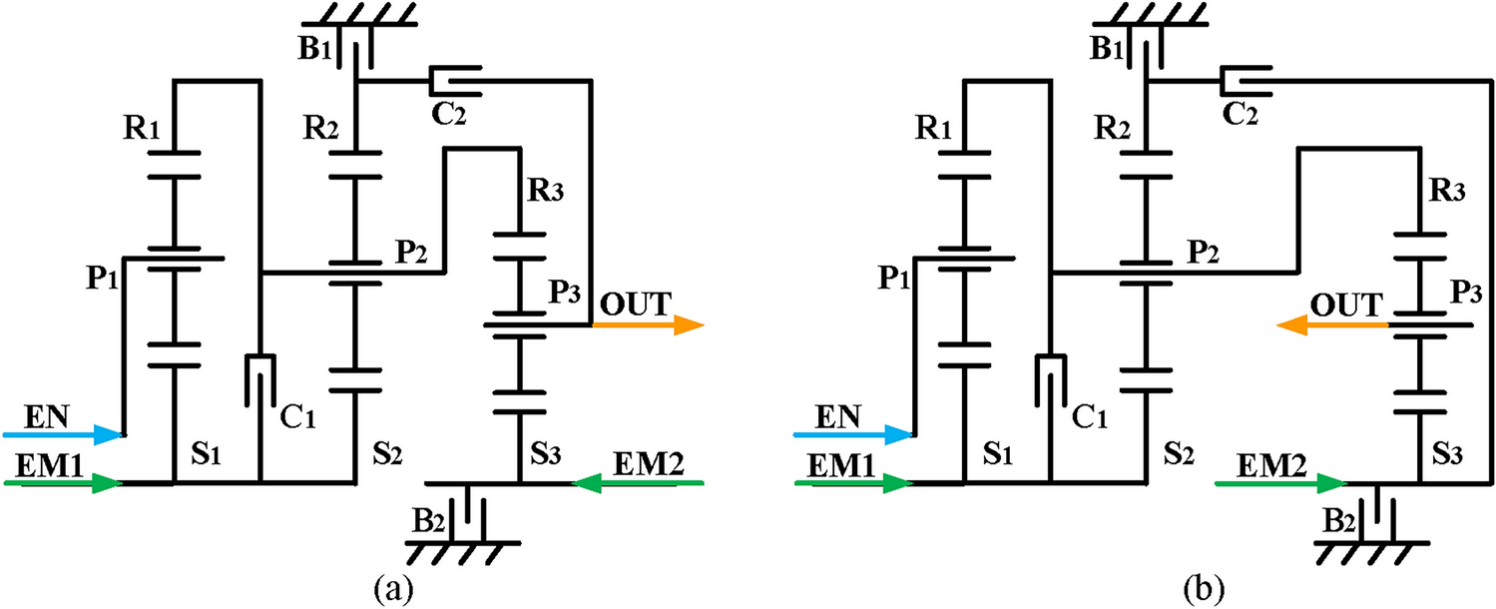
Two structure diagrams of two mode configurations synthesized from the input-split configuration of Fig. 13a.

Finally, two hybrid transmission configurations are obtained from the input-split configuration in Fig. 13a, and the lever models and structure diagrams are shown in Figs. 18 and 19 respectively. The configurations in Figs. 18b and 19b are the existing GM designs^16^.

**Fig. 20:**
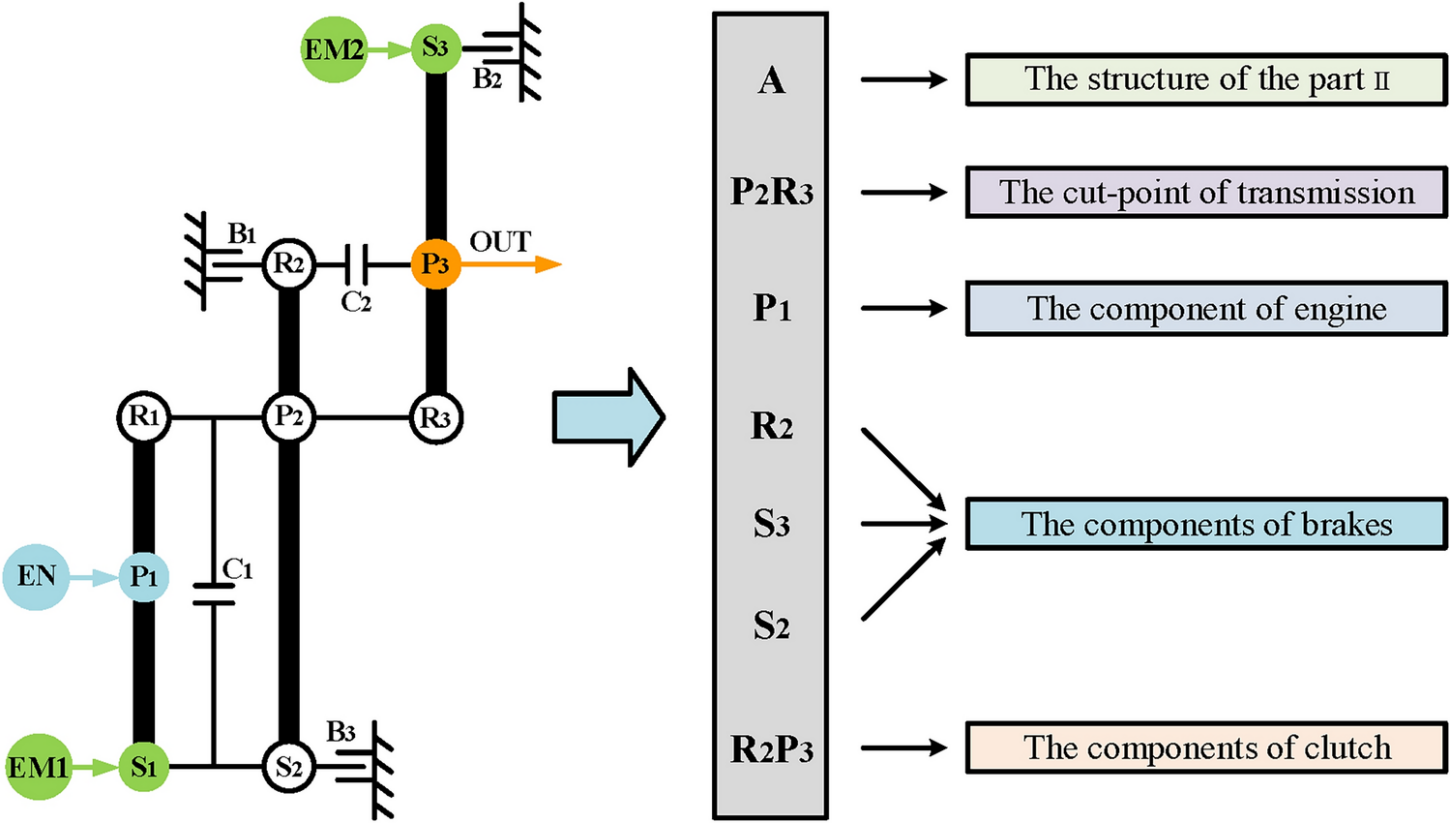
Configuration codes of the configuration of Fig. 18a.

The same synthesis process is carried out for the other nine substructures with two-PGT, and thirty-four two mode hybrid transmission configurations are obtained. In order to facilitate representation and further analysis, the configuration codes are used to represent these configurations. The configuration codes are mainly composed of configuration structure type, cut-link position, power sources position, and shifting element position. For the hybrid transmission in this paper, the EM1 and EM2 are placed on S_1_ and S_3_, respectively, the output shaft is placed on P_3_, and the clutch C_1_ is placed on the disconnected components of part II. Therefore, these elements are not included in the configuration code. Take the hybrid transmission configuration in Fig. 18a as an example, Fig. 20 shows its configuration code “A-P_2_R_3_-P_1_-R_2_-S_3_-S_2_-R_2_P_3_”. Table 2 shows the configuration codes of those thirty-four hybrid transmission configurations, ten of them (blue fonts) have appeared in reference^16^, which also confirms the effectiveness of this method. In other words, twenty-four new hybrid transmission configurations with three-PGT are synthesized using the combination of simple PGTs.

**Table 2:** The configuration codes of the hybrid transmissions with three-PGT.

The structure of part II	Configuration codes
1	A-R_2_S_3_-R_1_-R_3_-R_2_-S_2_R_3_, A-P_2_R_3_-P_1_-R_2_-S_3_-S_2_-R_2_S_3_, A-P_2_R_3_-P_1_-R_2_-S_3_-S_2_-R_2_P_3_
2	B-R_2_S_3_-P_1_-R_3_-R_1_-S_2_-P_2_R_3_, B-S_2_R_3_-P_1_-R_1_-S_3_-S_2_-R_2_P_3_
3	C-P_2_R_3_-P_1_-S_2_-S_3_-R_2_-S_2_P_3_, C-P_2_R_3_-P_1_-S_2_-S_3_-R_2_-S_2_S_3_, C-P_2_R_3_-P_1_-R_1_-S_3_-S_1_-S_1_S_3_
4	D-S_2_S_3_-P_1_-R_3_-R_1_-R_2_-P_2_R_3_, D-R_2_R_3_-P_1_-R_1_-S_3_-R_2_-S_2_P_3_
5	E-S_2_R_3_-P_1_-R_3_-R_1_-S_1_-R_2_P_3_
6	F-R_2_S_3_-P_1_-R_3_-R_1_-S_2_R_3_, F-P_2_R_3_-R_1_-R_2_-S_3_-R_3_-R_2_P_3_
7	G-S_2_S_3_-R_1_-R_3_-S_2_-P_2_P_3_, G-S_2_S_3_-R_1_-R_3_-S_2_-R_2_R_3_, G-R_2_S_3_-P_1_-R_3_-R_2_-S_2_P_3_ G-R_2_P_3_-P_1_-R_3_-S_1_-R_1_-S_2_S_3_, G-P_2_R_3_-R_1_-S_2_-S_3_-R_2_-S_2_P_3_, G-P_2_P_3_-R_1_-R_3_-S_2_-R_2_-S_2_S_3_
8	H-R_2_S_3_-R_1_-R_3_-R_2_-S_1_-S_2_P_3_, H-R_2_S_3_-R_1_-R_3_-R_2_-S_1_-S_2_R_3_, H-P_2_R_3_-P_1_-R_2_-S_3_-S_1_-R_2_P_3_ H-P_2_R_3_-P_1_-R_2_-S_3_-S_1_-R_2_S_3_, H-S_2_R_3_-R_1_-R_2_-S_3_-R_3_-R_2_P_3_, H-S_2_R_3_-R_1_-R_2_-R_3_-S_1_-R_2_S_3_ H-S_2_P_3_-R_1_-R_2_-R_3_-S_1_-R_2_S_3_, H-R_2_S_3_-P_1_-R_2_-R_3_-S_1_-S_2_R_3_, H-R_2_S_3_-P_1_-R_3_-R_2_-S_1_-P_2_R_3_
9	I-S_2_P_3_-R_1_-S_3_-R_2_-R_3_-P_2_R_3_
10	J-P_2_R_3_-P_1_-S_2_-S_3_-S_1_-S_2_P_3_, J-P_2_R_3_-P_1_-S_2_-S_3_-S_1_-S_2_S_3_, J-R_2_R_3_-R_1_-S_2_-S_3_-S_1_-S_2_P_3_ J-P_2_P_3_-P_1_-S_2_-S_3_-S_1_-R_2_R_3_, J-R_2_P_3_-R_1_-R_3_-S_2_-S_1_-S_2_S_3_

## Configuration synthesis of hybrid transmission with two-PGT

This synthesis method can also be applied to the hybrid transmission with two-PGT, and the synthesis process is the same as the previous hybrid transmission with three-PGT, but it is simpler. Therefore, the synthesis process of hybrid transmission with two-PGT is briefly introduced here.

Firstly, for the configuration of hybrid transmission with two-PGT, it can be divided into two substructures with one PGT, a simple PGT is satisfied. In order to distinguish the two substructures, they are called the first PGT and the second PGT according to their sequence. The constraints on electric motors, engine, and output shaft are the same as above. Therefore, the EM1, EM2, and output shaft are placed on the S_1_, S_2,_ and P_2_, respectively.

Secondly, realize electric motor alone mode. Similarly, the configuration shown in Fig. 8e is used as the design reference. Therefore, the electric motor alone mode configuration in a hybrid transmission system with two PGTs can be obtained as in Fig. 4b.

**Fig. 21:**
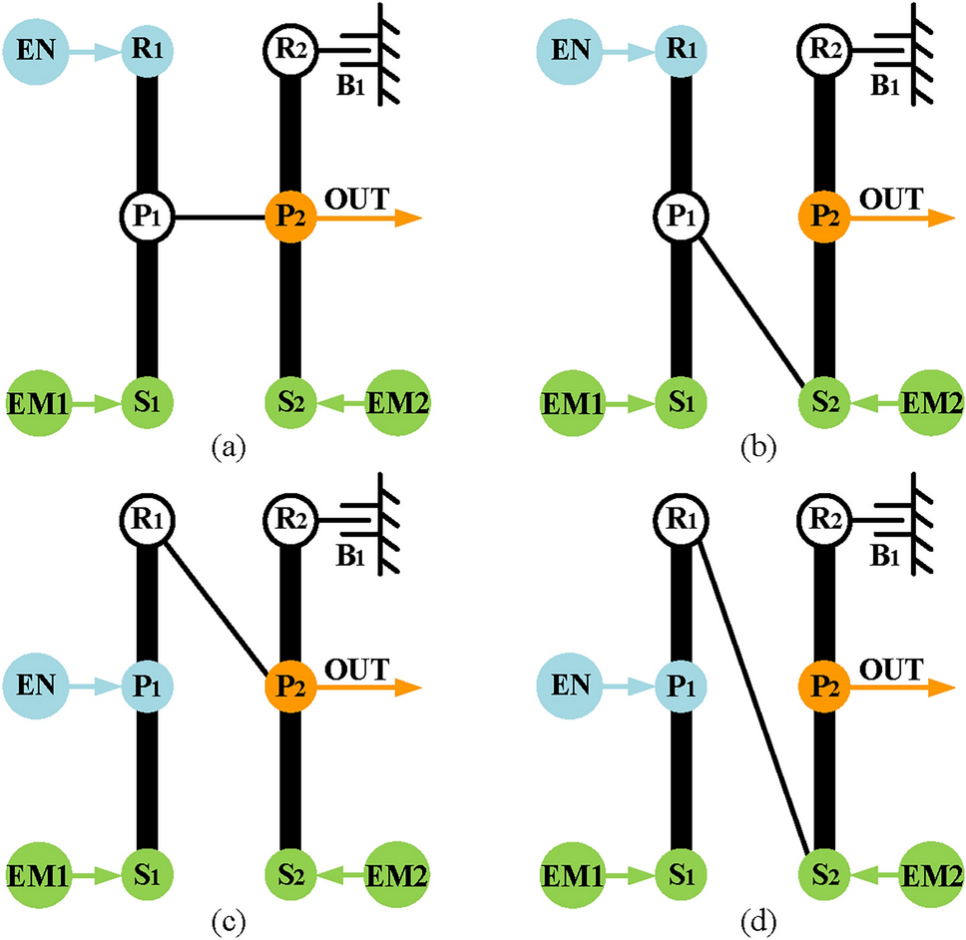
The four feasible input-split configurations with two-PGT.

Thirdly, realize input-split mode. The power split function can be achieved by the first PGT, and the second PGT is used to provide a fixed speed ratio. Therefore, it only needs to connect the output of the first PGT shown in Fig. 4b and a component of the substructure shown in Fig. 8e. It should be noted that when the output shaft (namely P_1_ or R_1_) of the first PGT is connected to the component with brake B_1_, the B_1_ cannot be transferred to the first PGT because the engine and the EM1 already exist in the first PGT, there is no component to place B_1_. As a result, four feasible configurations of input-split mode are obtained as shown in Fig. 21.

**Fig. 22:**
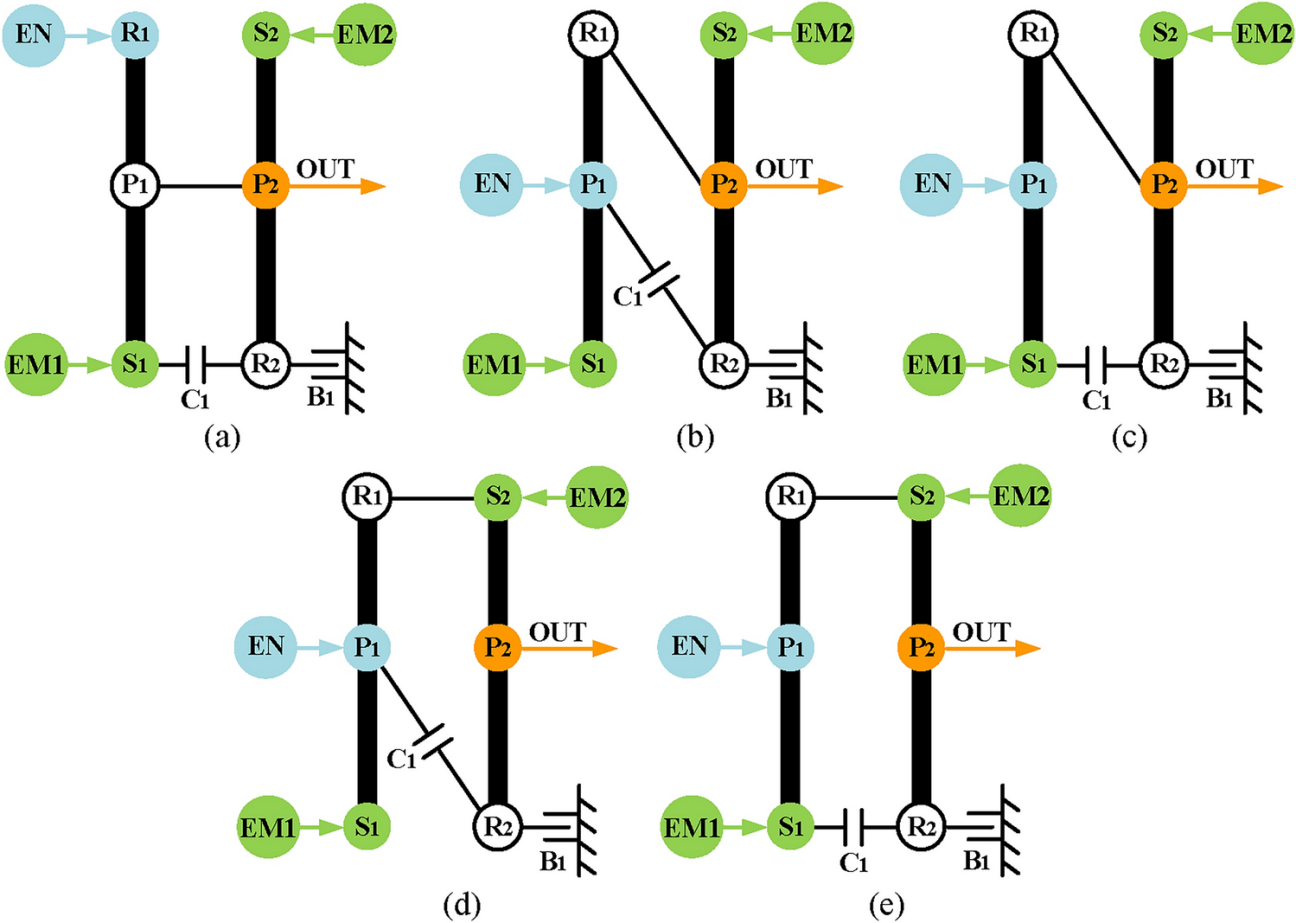
The five feasible compound-split configurations with two-PGT.

Fourthly, realize compound-split mode. This mode can be achieved by adding a clutch C_1_ to the configurations of the input-split mode. As for the arrangement of C_1_, in order to reduce the structural complexity, the C_1_ is specified to be added between the components of the first PGT and the second PGT. Since the compound-split mode configuration in Fig. 7c is taken as the ideal configuration in this paper, there are five feasible ways to add C_1_ for the input-split configurations in Fig. 21, as shown in Fig. 22.

**Fig. 23:**
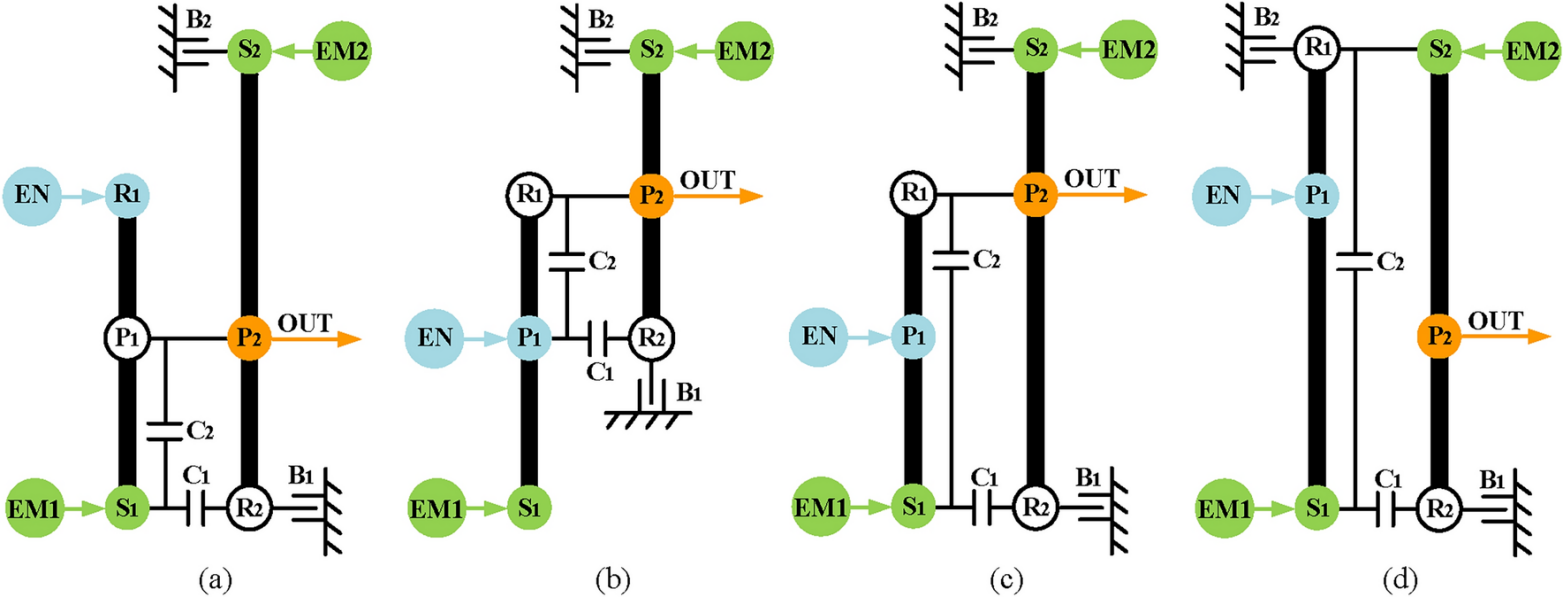
Four lever models of feasible two mode configurations with two-PGT.

**Fig. 24:**
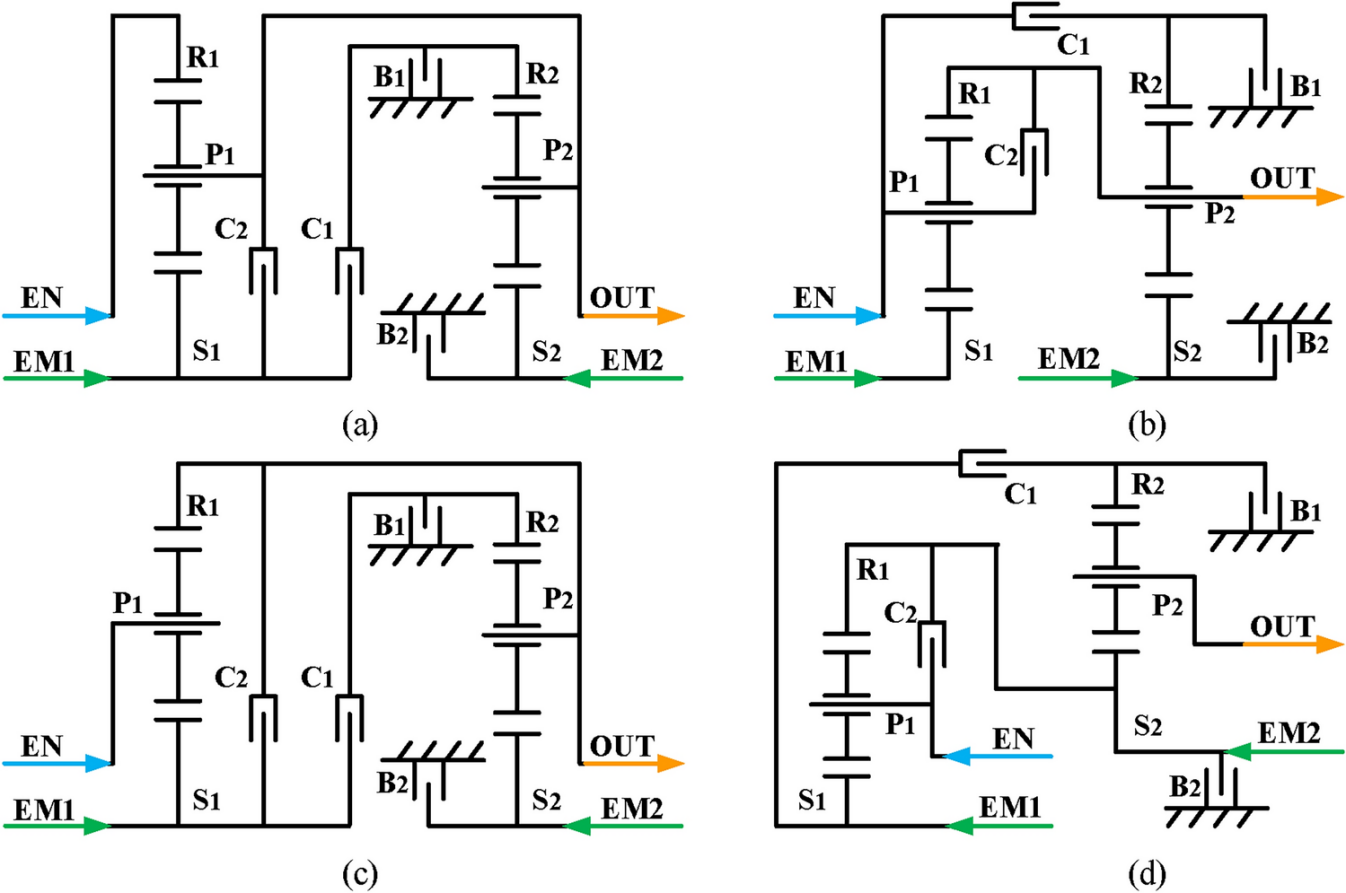
Four structure diagrams of feasible two mode configurations with two-PGT.

Finally, based on the previous analysis, engine alone mode can be achieved by removing the EM1 and EM2 and adding brakes and clutches to achieve. However, unlike the previous constraints of hybrid transmission with three-PGT, for a hybrid transmission with two-PGT, the number of brakes is no more than two. Consequently, four two mode hybrid transmission configurations are obtained after adding a clutch C_2_ and a brake B_2_, and the lever models and structure diagrams are shown in Figs. 23 and 24, respectively. The configurations shown in Figs. 23d and 24d can realize four fixed speed ratios and the other three can achieve three fixed speed ratios. The clutching sequence of configuration in Fig. 23d is shown in Table 3.

**Table 3:** The clutching sequence of the hybrid transmissions in Fig. 24(d).

Modes	B_1_	B_2_	C_1_	C_2_	Comments
EV	●				EM2 alone work
Input-split	●				EVT
Compound-split			●		EVT
1st FG	●			●	Underdrive
2nd FG	●		●		Underdrive
3rt FG			●	●	Direct drive
4th FG		●	●		Overdrive

The hybrid transmission configuration synthesized by this method has two hybrid power modes of input-split and compound-split. When the vehicle’s speed is relatively low, the input-split mode and large speed ratio are used to drive the vehicle, which reduces the torque requirement for the electric motors. When the vehicle speed is relatively high, the compound-split mode and small speed ratio are used to drive the vehicle, which reduces the speed requirement for the electric motors. Therefore, the two mode hybrid power system can reduce the peak power of the electric motors, making the motor smaller in size and smaller in mass. Moreover, these two mode hybrid systems add three or four fixed speed ratios, which allow the hybrid transmission to exit the hybrid mode and enter the fixed gear mode once the electric motor overheats, giving the hybrid transmission the same traction as a conventional automatic transmission.

## Analysis of new two mode hybrid transmission configurations

In this section, the kinematic analysis and torque analysis of a new two mode hybrid transmission configuration will be performed, and the expressions of the relationship between the speed and torque of the output shaft and the speed and torque of the motors and engine is derived, which provides reference for the subsequent configuration optimization. Taking the configuration shown in Fig. 24d as an example, its clutching sequence is shown in Table 3. This paper uses the method of establishing a three-dimensional model for simulation experiments to verify the feasibility of the scheme. The specific simulation settings are shown in Appendix A in Supplementary material.

### Electric motor alone mode

The kinematic analysis and torque analysis in this paper are carried out without considering the torque loss. According to the basic kinematics equations of PGT, we have:

1 $$\omega_{si} - (K_{i} + 1)\omega_{pi} + K_{i} \omega_{ri} = 0$$

where, $\omega_{si}$, $\omega_{pi}$, $\omega_{ri}$, represent the angular velocity of the sun gear, planetary carrier, and ring gear of the *i*-th PGT, respectively. The *K*_*i*_ denotes the characteristic parameters of the *i*-th PGT. Moreover, $\omega_{E}$, $T_{E}$, $\omega_{O}$, $T_{O}$, $\omega_{1}$, $T_{1}$ and $\omega_{2}$, $T_{2}$, are used to represent the angular velocities and torque of the engine, output shaft, EM1, and EM2 in this paper, respectively.

In the electric motor alone mode, the brake B_1_ is engaged and the R_2_ is fixed. The EM2 operates as a motor and drives the output shaft alone. Therefore, the relationship between the output and input angular velocity of the transmission can be derived as:

2 $$\left\{ {\begin{array}{*{20}l} {\omega _{{s2}} - (K_{2} + 1)\omega _{{p2}} + K_{2} \omega _{{r2}} = 0} \hfill \\ {\omega _{{r2}} = 0} \hfill \\ {\omega _{{s2}} = \omega _{2} } \hfill \\ \end{array} } \right.$$

The angular velocity of the output shaft can be obtained by solving Eq. (2), therefore,

3 $$\omega_{O} = \omega_{p2} = \frac{{\omega_{2} }}{{K_{2} + 1}}$$

**Fig. 25:**
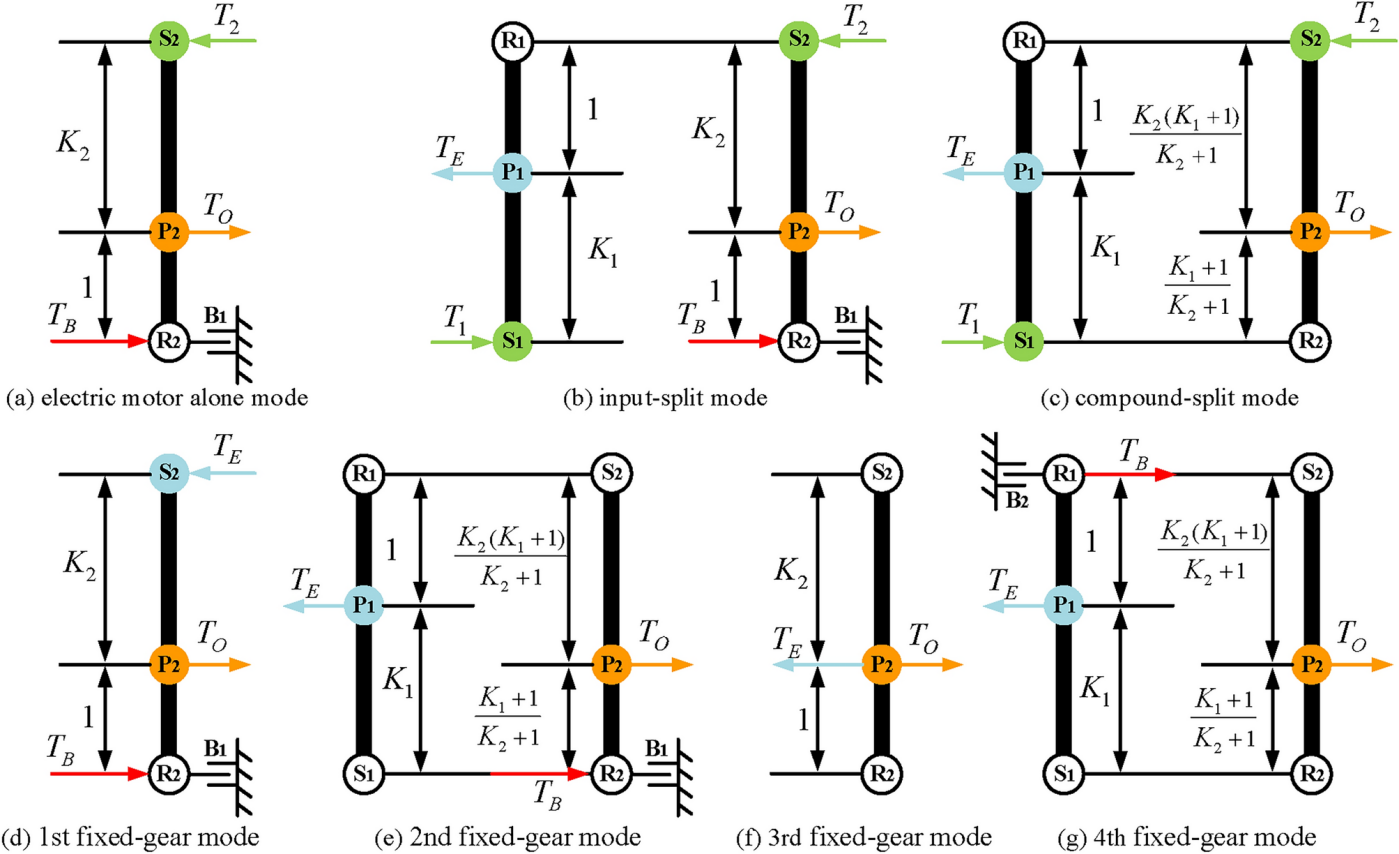
lever analogy of each operation mode of Fig. 24d.

As for the torque analysis, the analogy lever method^37^ is used to determine the torque of the modes. As shown in Fig. 25a, where, *T*_*B*_ represents the torque of the brake. The torque of the output shaft in electric motor alone mode can be obtained by the balance of torque in Fig. 25a, therefore,

4 $$T_{O} = (K_{2} + 1)T_{2}$$

### Input-split mode

In input-split mode, the brake B_1_ is engaged, and the EM1 is used as a generator to generate electric power. Based on Eq. (1), the relationship between the output and input angular velocity of the transmission can be derived as:

5 $$\left\{ {\begin{array}{*{20}l} {\omega _{{s1}} - (K_{1} + 1)\omega _{{p1}} + K_{1} \omega _{{r1}} = 0} \hfill \\ {\omega _{{s2}} - (K_{2} + 1)\omega _{{p2}} + K_{2} \omega _{{r2}} = 0} \hfill \\ {\omega _{{s1}} = \omega _{1} } \hfill \\ {\omega _{{p1}} = \omega _{E} } \hfill \\ {\omega _{{r1}} = \omega _{{s2}} } \hfill \\ {\omega _{{r2}} = 0} \hfill \\ \end{array} } \right.$$

The angular velocity of the output shaft can be obtained by solving Eq. (5), therefore,

6 $$\omega_{O} = \omega_{p2} = \frac{{(K_{1} + 1)\omega_{E} - \omega_{1} }}{{K_{1} (K_{2} + 1)}}$$

Based on the torque balance of the sun gear (S_1_) of the first PGT and the ring gear (R_2_) of the second PGT in Fig. 25b, the torque relationship between the engine, EM1and EM2 can be written as:

7 $$\left\{ {\begin{array}{*{20}l} {T_{E} K_{1} - T_{{r1}} (K_{1} + 1) = 0} \hfill \\ {T_{{r1}} - T_{{s2}} = 0} \hfill \\ {T_{O} - (T_{{s2}} + T_{2} )(K_{2} + 1) = 0} \hfill \\ \end{array} } \right.$$

where *T*_*r1*_ and *T*_*s2*_ are the torque of the ring gear of the first PGT and the sun gear of the second PGT respectively. Therefore, the torque of the output shaft in input-split mode can be obtained by solving Eq. (7).

8 $$T_{O} = (K_{2} + 1)T_{2} + \frac{{K_{1} (K_{2} + 1)}}{{K_{1} + 1}}T_{E}$$

### Compound-split mode

In compound-split mode, the clutch C_1_ is engaged. Based on Eq. (1), the relationship between the output and input angular velocity of the transmission can be derived as:

9 $$\left\{ {\begin{array}{*{20}l} {\omega _{{s1}} - (K_{1} + 1)\omega _{{p1}} + K_{1} \omega _{{r1}} = 0} \hfill \\ {\omega _{{s2}} - (K_{2} + 1)\omega _{{p2}} + K_{2} \omega _{{r2}} = 0} \hfill \\ {\omega _{{s1}} = \omega _{1} } \hfill \\ {\omega _{{p1}} = \omega _{E} } \hfill \\ {\omega _{{r1}} = \omega _{{s2}} } \hfill \\ {\omega _{{r2}} = \omega _{{s1}} } \hfill \\ \end{array} } \right.$$

The angular velocity of the output shaft can be obtained by solving Eq. (8), therefore,

10 $$\omega_{O} = \omega_{p2} = \frac{{(K_{1} K_{2} - 1)\omega_{1} + (K_{1} + 1)\omega_{E} }}{{K_{1} (K_{2} + 1)}}$$

Based on the torque balance of the planetary gear carrier (P_1_) of the first PGT in Fig. 25c, the torque relationship between the output, EM1and EM2 can be written as:

11 $$T_{O} \left( {K_{1} - \frac{{K_{1} + 1}}{{K_{2} + 1}}} \right) + T_{2} + T_{1} K_{1} = 0$$

Therefore, the torque of the output shaft in input-split mode can be obtained by solving Eq. (11).

12 $$T_{O} = - (T_{2} + K_{1} T_{1} )\frac{{K_{2} + 1}}{{K_{1} K_{2} - 1}}$$

### The first fixed gear

In the first fixed gear, the brake B_1_ and the clutch C_2_ are engaged. Based on Eq. (1), the relationship between the output and input angular velocity of the transmission can be derived as:

13 $$\left\{ {\begin{array}{*{20}l} {\omega _{{s1}} - (K_{1} + 1)\omega _{{p1}} + K_{1} \omega _{{r1}} = 0} \hfill \\ {\omega _{{s2}} - (K_{2} + 1)\omega _{{p2}} + K_{2} \omega _{{r2}} = 0} \hfill \\ {\omega _{{p1}} = \omega _{E} } \hfill \\ {\omega _{{r1}} = \omega _{{s2}} } \hfill \\ {\omega _{{r2}} = 0} \hfill \\ \end{array} } \right.$$

The angular velocity of the output shaft can be obtained by solving Eq. (13), therefore,

14 $$\omega_{O} = \omega_{p2} = \frac{{\omega_{E} }}{{(K_{2} + 1)}}$$

The torque of the output shaft in the first fixed gear can be obtained by the torque balance in Fig. 25d.

15 $$T_{O} = (K_{2} + 1)T_{E}$$

### The second fixed gear

In the second fixed gear, the brake B_1_ and the clutch C_1_ are engaged. Based on Eq. (1), the relationship between the output and input angular velocity of the transmission can be derived as:

16 $${\begin{array}{*{20}l} {\omega _{{s1}} - (K_{1} + 1)\omega _{{p1}} + K_{1} \omega _{{r1}} = 0} \hfill \\ {\omega _{{s2}} - (K_{2} + 1)\omega _{{p2}} + K_{2} \omega _{{r2}} = 0} \hfill \\ {\omega _{{p1}} = \omega _{E} } \hfill \\ {\omega _{{r2}} = \omega _{{s1}} } \hfill \\ {\omega _{{r1}} = \omega _{{s2}} } \hfill \\ {\omega _{{r2}} = 0} \hfill \\ \end{array} }$$

The angular velocity of the output shaft can be obtained by solving Eq. (16), therefore,

17 $$\omega_{O} = \omega_{p2} = \frac{{(K_{1} + 1)\omega_{E} }}{{K_{1} (K_{2} + 1)}}$$

The torque of the output shaft in the second fixed gear can be obtained by the balance of torque in Fig. 25e.

18 $$T_{O} = \frac{{K_{1} (K_{2} + 1)}}{{K_{1} + 1}}T_{E}$$

### The third fixed gear

In the third fixed gear, the clutch C_1_ and the clutch C_2_ are engaged. Based on Eq. (1), the relationship between the output and input angular velocity of the transmission can be derived as:

19 $$\left\{ {\begin{array}{*{20}l} {\omega _{{s1}} - (K_{1} + 1)\omega _{{p1}} + K_{1} \omega _{{r1}} = 0} \hfill \\ {\omega _{{s2}} - (K_{2} + 1)\omega _{{p2}} + K_{2} \omega _{{r2}} = 0} \hfill \\ {\omega _{{p1}} = \omega _{E} } \hfill \\ {\omega _{{{\text{r2}}}} = \omega _{{s1}} } \hfill \\ {\omega _{{r1}} = \omega _{{s1}} } \hfill \\ \end{array} } \right.$$

The angular velocity of the output shaft can be obtained by solving Eq. 19, therefore,

20 $$\omega_{O} = \omega_{p2} = \omega_{E}$$

The torque of the output shaft in the third fixed gear can be obtained by the balance of torque in Fig. 25f.

21 $$T_{O} = T_{E}$$

### The fourth fixed gear

In the fourth fixed gear, the brake B_2_ and the clutch C_1_ are engaged. Based on Eq. (1), the relationship between the output and input angular velocity of the transmission can be derived as:

22 $$\left\{ {\begin{array}{*{20}l} {\omega _{{s1}} - (K_{1} + 1)\omega _{{p1}} + K_{1} \omega _{{r1}} = 0} \hfill \\ {\omega _{{s2}} - (K_{2} + 1)\omega _{{p2}} + K_{2} \omega _{{r2}} = 0} \hfill \\ {\omega _{{p1}} = \omega _{E} } \hfill \\ {\omega _{{r2}} = \omega _{{s1}} } \hfill \\ {\omega _{{r1}} = \omega _{{s2}} } \hfill \\ {\omega _{{r1}} = 0} \hfill \\ \end{array} } \right.$$

The angular velocity of the output shaft can be obtained by solving Eq. (22), therefore,

23 $$\omega_{O} = \omega_{p2} = \frac{{K_{2} (K_{1} + 1)\omega_{E} }}{{K_{2} + 1}}$$

The torque of the output shaft in the fourth fixed gear can be obtained by the torque balance in Fig. 25g.

24 $$T_{O} = \frac{{(K_{2} + 1)T_{E} }}{{K_{2} (K_{1} + 1)}}$$

## Conclusion

A complete and efficient configuration synthesis method will improve the efficiency of hybrid transmission design and create more feasible novel configurations. In this paper, a new method is proposed to synthesize the configuration of two mode hybrid transmissions. The hybrid transmission structure is divided into two simple PGTs, and these substructures, power sources, and shifting elements are combined to realize the electric alone mode, input-split mode, compound-split mode, and engine alone mode in turn. As a result, 38 feasible two mode configurations, including GM designs and new configurations, are obtained. The synthesis results could serve as a configuration foundation for energy management strategy, mode switching, and software control in the future. Furthermore, this method can be used to synthesize hybrid transmissions with multiple PGTs and different operation modes. However, the characteristic parameters of PGTs and the working characteristics of engine and motor are not considered in the synthesis method in this paper. Therefore, how to take these parameters and characteristics into account is the future research direction to improve the synthesis method of this paper.

## Supplementary Information

Supplementary Information.

## Declarations

### Data availability

The datasets used and/or analysed during the current study available from the corresponding author on reasonable request.
