# Supplementary material for: Powertrain configuration design for two mode power split hybrid electric vehicle
Source: Sci Rep. 2025 Jan 29;15:3676. doi: 10.1038/s41598-025-87378-w (PMC11779879; doi:10.1038/s41598-025-87378-w)
Supplement: Supplementary file 1 — Supplementary Information. [file 41598_2025_87378_MOESM1_ESM.pdf]

Appendix A

we have conducted a preliminary verification by developing a three-dimensional model (created by the first aut hor Ke Tao using SolidWorks 2022 (version: 2022 SP3.1, license number: 00180000 0010 9647 NKHW WBH 3), and the author owns the ownership of the figure) for simulation purposes as shown in Fig.A1.

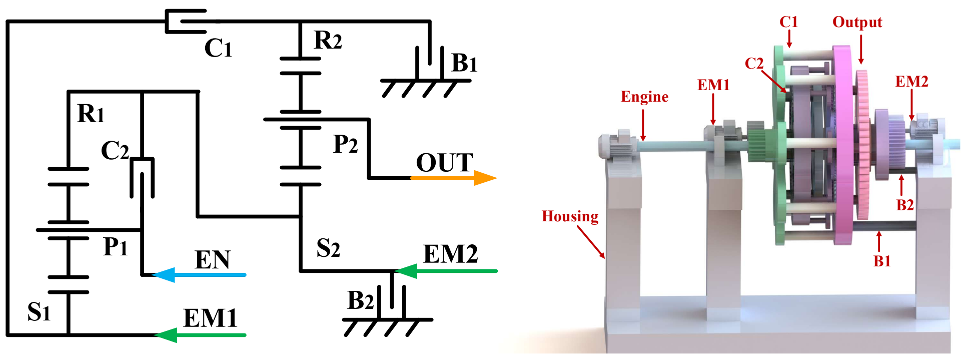

(a) structure diagram (b) simulation mode

Figure A1. A two-mode hybrid configuration (a) and (b) its simulation verification model (created by the first author Ke Tao using SolidWorks 2022 (version: 2022 SP3.1, license number: 00180000 0010 9647 NKHW WBH3), and the author owns the ownership of the figure)

In the simulation settings, a fixed speed torque output motor was specifically set as the power source to simulate the operating state of the engine in the optimal working range. Through simulation experiments, observe the speed torque relationship between the dual motors and the output shaft in power split mode of this configuration, verify whether the expected speed torque coupling relationship can be achieved between each power source, and thus verify the practical feasibility of this configuration.

Mode 1: Electric motor alone mode. In this mode, the brake B1 is engaged and the R2 is fixed. The EM2 operates as a motor and drives the output shaft alone. Therefore, in the simulation experiment, the speed and torque settings for EM 2 are as follows:

$$\begin{cases} \omega_2 = 300\cos(t) \\ T_2 = 100\cos(t) + 100 \end{cases} \tag{B1}$$

The total duration of the simulation experiment is set to 10 seconds, and the final simulation results of Mode 1 are shown in Fig. A2.

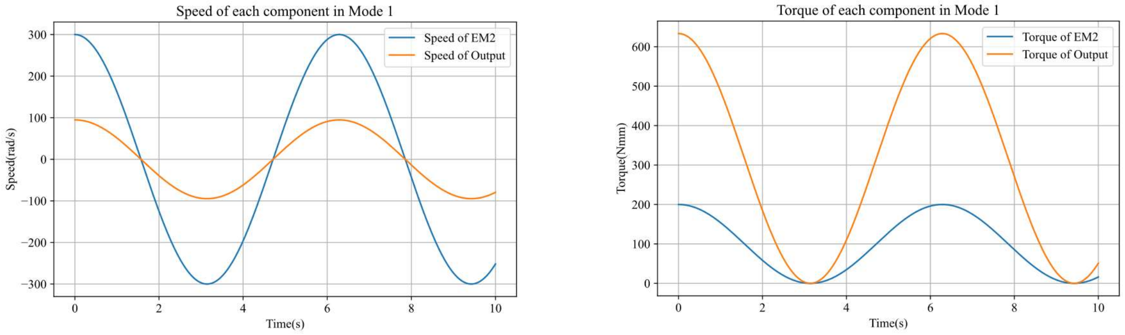

Figure. A2. Speed and torque between the EM2 and output in Mode 1.

Mode 2: input-split mode. In this mode, the brake B1 is engaged, and the EM1 is used as a generator to generate electric power. To simulate the efficient operation of the engine, set the engine speed to a constant value of 300 °/s and fix the torque to 100 Nmm. The speed at the motor and the impedance torque at the output shaft are set as follows:

$$\begin{cases} \omega_2 = 300\cos(t) \\ T_O = 100\cos(t) + 150 \end{cases} \tag{B2}$$

The total duration of the simulation experiment is set to 10 seconds, and the final simulation results of Mode 2 are shown in Fig. A3.

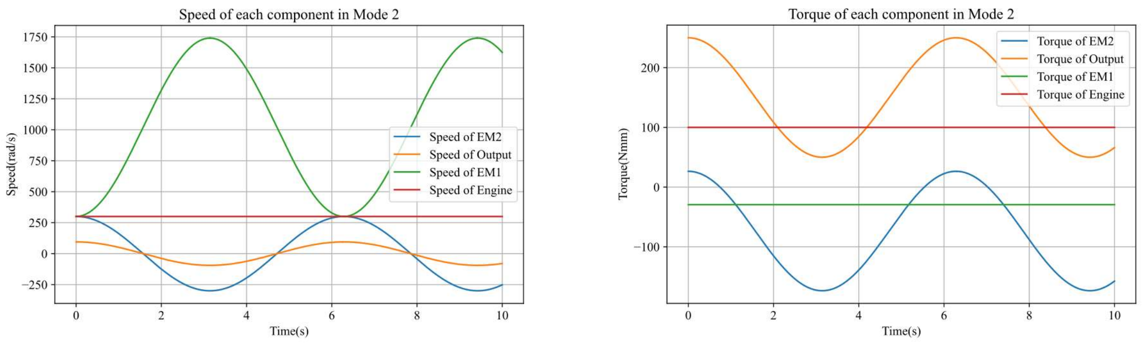

Figure. A3. The speed and torque of each component in Mode 2.

Mode 3: compound-split mode. Due to the lack of a clear distinction between the generator and the motor in the compound shunt mode, it will change with the operating conditions. In the simulation experiment of this section, the clutch C1 is engaged and the engine input speed is set to a constant value of 300 °/s, while the fixed torque is 100Nmm. The speed at the EM2 and the impedance torque at the output shaft are set as follows:

$$\begin{cases} \omega_2 = 300\cos(t) \\ T_O = 100\cos(t) + 100 \end{cases} \tag{B3}$$

The total duration of the simulation experiment is set to 10 seconds, and the final simulation results of Mode 3 are shown in Fig. A4.

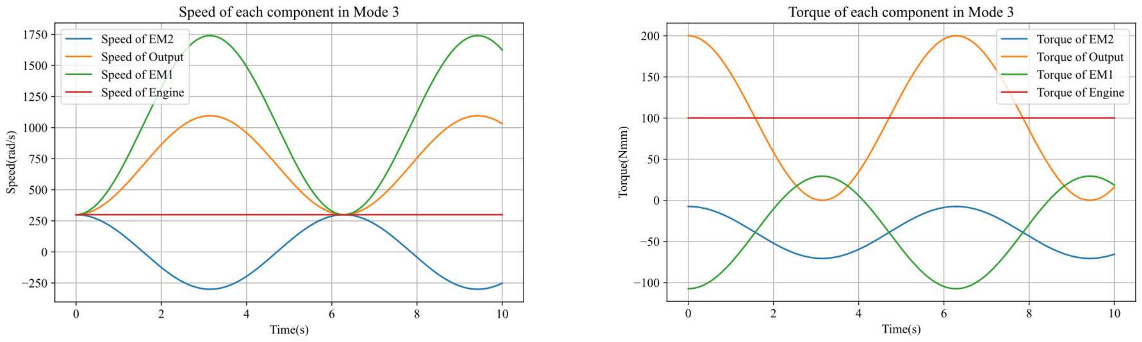

Figure. A4. The speed and torque of each component in Mode 3.

Mode 4-7: The fixed gears. In these modes, the two motors do not input power, only the engine inputs power. Set the engine speed and torque as follows in the simulation settings:

$$\begin{cases} \omega_{EN} = 300\cos(t) \\ T_{EN} = 100\cos(t) \end{cases} \tag{B4}$$

The total duration of the simulation experiment is set to 10 seconds, and the final simulation results of Mode 4-7 are shown in Fig. A5.

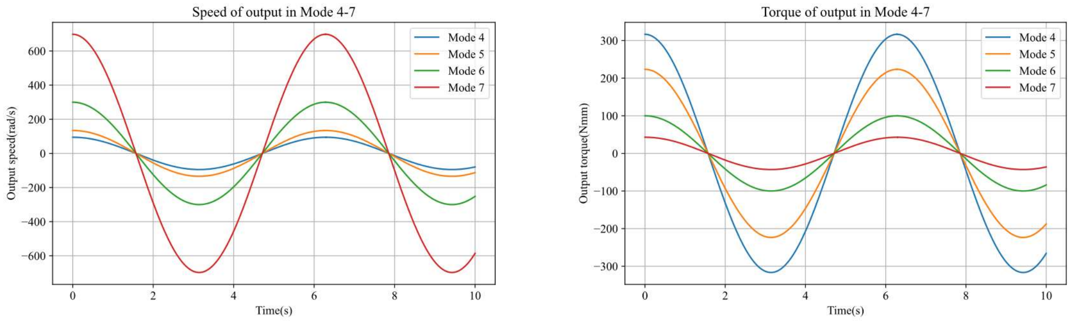

Figure. A5. The speed and torque of output in Mode 4-7.
